# Ionic poly(dimethylsiloxane)–silica nanocomposites: Dispersion and self-healing

**DOI:** 10.1557/s43577-022-00346-x

**Published:** 2022-09-16

**Authors:** Clément Mugemana, Ahmad Moghimikheirabadi, Didier Arl, Frédéric Addiego, Daniel F. Schmidt, Martin Kröger, Argyrios V. Karatrantos

**Affiliations:** 1 Materials Research and Technology, Luxembourg Institute of Science and Technology, Esch-sur-Alzette, Luxembourg; 2 Polymer Physics, Department of Materials, ETH Zürich, Zurich, Switzerland

**Keywords:** Composite, Interface, Nanoscale, Inorganic and simulation

## Abstract

**Abstract:**

Poly(dimethylsiloxane) (PDMS)-based nanocomposites have attracted increasing attention due to their inherent outstanding properties. Nevertheless, the realization of high levels of dispersion of nanosilicas in PDMS represents a challenge arising from the poor compatibility between the two components. Herein, we explore the use of ionic interactions located at the interface between silica and a PDMS matrix by combining anionic sulfonate-functionalized silica and cationic ammonium-functionalized PDMS. A library of ionic PDMS nanocomposites was synthesized and characterized to highlight the impact of charge location, density, and molecular weight of ionic PDMS polymers on the dispersion of nanosilicas and the resulting mechanical reinforcement. The use of reversible ionic interactions at the interface of nanoparticles–polymer matrix enables the healing of scratches applied to the surface of the nanocomposites. Molecular dynamics simulations were used to estimate the survival probability of ionic cross-links between nanoparticles and the polymer matrix, revealing a dependence on polymer charge density.

**Impact statement:**

Poly(dimethylsiloxane) (PDMS) has been widely used in diverse applications due to its inherent attractive and multifunctional properties including optical transparency, high flexibility, and biocompatibility. The combination of such properties in a single polymer matrix has paved the way toward a wide range of applications in sensors, electronics, and biomedical devices. As a liquid at room temperature, the cross-linking of the PDMS turns the system into a mechanically stable elastomer for several applications. Nanofillers have served as a reinforcing agent to design PDMS nanocomposites. However, due to significant incompatibility between silica and the PDMS matrix, the dispersion of nanosilica fillers has been challenging. One of the existing strategies to improve nanoparticle dispersion consists of grafting oppositely charged ionic functional groups to the nanoparticle surface and the polymer matrix, respectively, creating nanoparticle ionic materials. Here, this approach has been explored further to improve the dispersion of nanosilicas in a PDMS matrix. The designed ionic PDMS nanocomposites exhibit self-healing properties due to the reversible nature of ionic interactions. The developed synthetic approach can be transferred to other kinds of inorganic nanoparticles dispersed in a PDMS matrix, where dispersion at the nanometer scale is a prerequisite for specific applications such as encapsulants for light-emitting diodes (LEDs).

**Graphical abstract:**

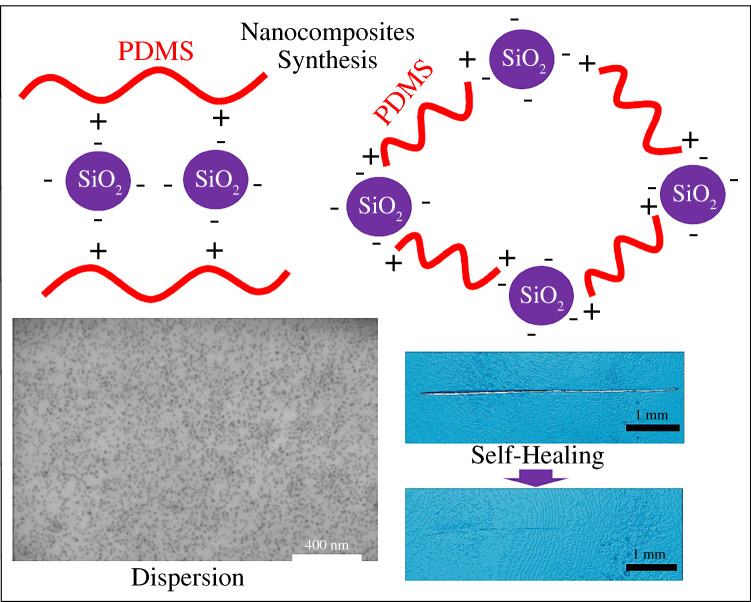

**Supplementary information:**

The online version contains supplementary material available at 10.1557/s43577-022-00346-x.

## Introduction

Polymer nanocomposites (PNCs) are most generally defined as polymers that contain a second phase with at least one nanoscale dimension—typically a nanoparticle component.^1,2^ In practice, the existence, study, and industrial exploitation of these materials predate the terminology now used to describe them.^3^ As an example, the ability of carbon black (arguably the earliest nanofiller) to reinforce rubber was discovered by Mote and colleagues in 1904, even before the existence of macromolecules was recognized. The commercialization of the first silicone rubbers took place in the mid-20th century, with their efficient reinforcement by silica nanoparticles resulting in a new generation of rubber nanocomposites—again, without the use of this terminology. In the 1980s, researchers at Toyota Central R&D showed that the incorporation of small quantities of high-aspect-ratio clay nanofillers in a polyamide 6 matrix resulted in enhanced stiffness, strength, and heat distortion temperature with no loss in ductility.^4^ This unexpected result in the field of filler thermoplastics (where embrittlement was the general result when rigid particles were added) led to the widespread use of the term “polymer nanocomposite” as well as an explosion of interest in nanoclay-based systems.^5^ Carbon nanotubes were the next class of nanofillers to gain significant attention in this context,^6^ followed by graphene^7^ and nanocellulose.^8^ Many other nanofillers have been studied besides, and the literature is populated by excellent reports and reviews too numerous to fully represent here. That said, in introducing this class of materials, we are nevertheless able to offer a few generalizations. For one thing, control of the dispersion state of nanoparticles in the polymer matrix is critical to realizing the desired properties. This is because nanocomposites are not simply the sum of their parts; when high levels of nanoparticle dispersion are achieved, the majority of the polymer chains are at or near an interface and display non-bulk behavior as a result. In order to realize high levels of nanoparticle dispersion in such systems, a common strategy is to modify the surface of nanoparticles via chemical treatment, polymer grafting, or compatibilizer addition to improve the interfacial interactions between nanoparticles and the polymer matrix.^9^ Proper processing is important as well, but in the absence of thermodynamic compatibility, stable dispersion and properties enhancements are not achieved in practice.

In this context, a plethora of polymer matrices have been studied. Returning to the topic of silicone rubbers, poly(dimethylsiloxane) (PDMS) is the most widely explored and utilized polysiloxane, possessing an extremely low glass transition (*T*_g_), excellent thermal stability, high permeability, and good biocompatibility.^10^ As it is a liquid at room temperature, most applications require PDMS to be chemically cross-linked and/or combined with nanofillers to realize the requisite mechanical properties. A plethora of nanofillers have been used for reinforcement of the PDMS matrix,^11^ including montmorillonite,^12^ carbon nanotubes,^13^ gold,^14^ and carbon fibers,^15^ for diverse applications such as electrically conductive elastomers,^16^ strain sensors,^17^ superhydrophobic coatings,^18^ and others. As alluded to above, fumed silica has been one of the most favored reinforcing fillers for silicones due to strong hydrogen-bond-mediated interactions between hydroxyl groups from the fumed silica surface and PDMS siloxane bonds.^19^ PDMS–silica nanocomposites were prepared using different approaches including the sol–gel process to generate nanosilica *in situ* within a preformed PDMS network, preventing aggregation.^20^ Solution mixing and melt blending techniques have also been employed for the formation of PDMS-silica nanocomposites. Although for other kinds of polymer matrices such as PMMA, weaker interactions have led to well-dispersed nanoparticles,^21^ it has been reported that for the PDMS matrix, dispersion is more challenging. Mechanical blending of vinyl-terminated PDMS and silyl-terminated PDMS in the presence of Karstedt’s catalyst has been used to disperse silica nanoparticles in a PDMS matrix.^22,23^ Centrifugal mixing,^24^ ultrasonication, and miniemulsion polymerization^25^ have also been utilized to improve the dispersion of silica clusters in PDMS. The grafting of PDMS to the silica nanoparticle surface to improve interactions with the PDMS matrix increases cross-link density and improves the mechanical properties.^26^ Finally, adding a catalyst to initiate the rupture of siloxane bonds has been explored to improve the dispersion of silica nanoparticles in a PDMS matrix as well.^27^

One element that unites the vast majority of the work on polymer nanocomposites, including the various reports concerning PDMS previously described, is the means by which the polymer and the nanofiller interact. By far, the most common approach is to rely on relatively weak secondary interactions—for instance, the formation of hydrogen bonds at the polymer–filler interface, entanglements between a grafted polymer chain and the polymer matrix. This is convenient from a processing standpoint, because the polymer phase and the (modified) nanoparticles may then be physically mixed to form the polymer nanocomposite, but can easily result in poor dispersion and properties if the interactions are not sufficiently favorable. The formation of strong covalent bonds between the polymer and the (modified) nanoparticle is also practiced, typically in nanocomposites produced via *in situ* polymerization. In this case, high levels of nanoparticle dispersion are favored as well as strong polymer–filler interactions. In addition to being less convenient from a processing standpoint, however, a second issue with this approach is that the interfacial interactions in such systems can be “too strong,” to the point of compromising toughness given the impossibility to accommodate any strain at the polymer/filler interface except through irreversible debonding or fracture.

In recognition of these limitations, a more recently explored strategy to realize high levels of nanoparticle dispersion and strong interfacial interactions while allowing for reversible interfacial debonding involves the use of ionic interactions. The combination of oppositely charged polymers and nanoparticles has led to nanocomposites displaying highly dispersed nanostructures^28–30^ based on poly(ethylene oxide),^31^ polylactide and poly(ε-caprolactone-co-d,l-lactide),^32,33^ and polyurethane.^34^ In keeping with these reports, recent simulations work on such systems highlights the potential for unique behavior and interesting mechanical performance as a function of ionic nanocomposite design.^35^

In addition to the promise of strong ionic interactions at the polymer–filler interface as a means of generating novel polymer nanocomposites, their reversible nature promises additional functionality related to self-healing behavior. Specific to PDMS networks, a number of self-healing systems have been studied, given that cross-linked PDMS networks are known for possessing mechanical properties similar to those of body tissues. The incorporation of reversible interactions (hydrogen bonds,^36–38^ ionic bonds,^39–41^ metal complexes,^42,43^ dynamic transesterification,^44^ etc.) into PDMS networks, cross-linked co-networks,^45^ and coatings^46^ provides tunable self-healing behavior while maintaining mechanical strength and high stretchability.

Building on the aforementioned bodies of knowledge, we report for the first time the use of ionic interactions to enhance the distribution and dispersion of nanosilica in a PDMS matrix by combining cationic ammonium-functionalized PDMS and anionic sulfonate-functionalized nanosilicas. The effects of PDMS molecular weight (*MW*), charge density, and charge location on nanosilica dispersion and mechanical reinforcement are investigated, and healing behavior in this context as well. Here, the use of reversible ionic interactions at the polymer–nanofiller interface is exploited not only for the purposes of stress transfer and reinforcement but also for triggering healing in scratched nanocomposite films. Finally, these in-depth experimental studies are complemented by coarse-grained molecular dynamics simulations of ionic nanocomposites to estimate the expected lifetimes of ionic cross-links between ionic nanoparticles and polymers, thus providing further insights into the origins of the observed behavior.

## Results and discussion

### Synthesis and characterization of ionic PDMS

**Figure 1:**
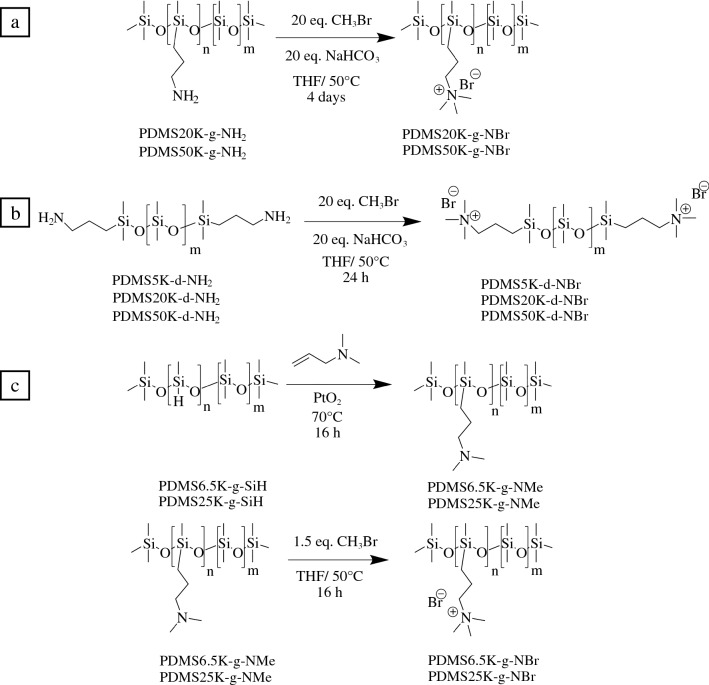
Synthesis of PDMS grafted with trimethylammonium bromide from poly(aminopropylmethylsiloxane-*r*-dimethylsiloxane) (a), synthesis of trimethylammonium bromide-terminated PDMS from aminopropyl-terminated polydimethysiloxane (b), and synthesis of the PDMS grafted with dimethylamine by hydrosilylation of poly(methylhydrosiloxane-*r*-dimethylsiloxane) and subsequent quaternization in the presence of methyl bromide (c).

A library of ionic PDMS melts with molar masses in the range of 6500–50,000 g/mol and trimethylammonium concentrations varying from 0.07 to 1.73 mmol/g were prepared either by a direct quaternization of (aminopropylmethylsiloxane-*r*-dimethylsiloxane) copolymers or hydrosilylation of (methylhydrosiloxane-*r*-dimethylsiloxane) copolymers in a two-step reaction (**Figure ****1**). The hydrosilylation of poly(methylhydrosiloxane-*r*-dimethylsiloxane) copolymers having molar masses of 6500 and 25,000 g/mol with respective concentrations of 0.87 and 0.69 mmol/g of methylhydrosiloxane was performed in the presence of *N,N*-dimethylallylamine and PtO_2_ catalyst to yield the PDMS6.5K-g-NMe and PDMS25K-g-NMe copolymers (Figure S1 in Supporting Information). The nomenclature of the samples was defined according to the molecular weight, functionality, and charge location, for example, PDMS6.5K-g-NBr: 6.5 kg/mol; g: grafted: NBr: trimethylammonium bromide. The quaternization reaction in the presence of methyl bromide was performed in a second step to give the trimethylammonium-functionalized PDMS corresponding to PDMS6.5K-g-NBr and PDMS25K-g-NBr PDMS copolymers (Figure S2). The direct quaternization of poly(aminopropylmethylsiloxane-*r*-dimethylsiloxane) copolymers having molar masses of 20,000 (PDMS20K-g-NBr) and 50,000 (PDMS50K-g-NBr) g/mol with respective cationic charge densities of 1.73 and 0.83 mmol/g was achieved in the presence of a large excess of methyl bromide and sodium bicarbonate in THF over four days (Figure S3). The impact of charge location on nanosilica dispersion was investigated by functionalizing the chain ends of PDMS with trimethylammonium from aminopropyl-terminated PDMS having molar masses of 5000, 25,000 and 50,000 g/mol to yield PDMS5K-d-NBr, PDMS25K-d-NBr, and PDMS50K-d-NBr, respectively (Figure S4).

The synthesized ionic polymers were characterized by DSC to determine the impact of cationic pendant group location and concentration on the glass-transition temperature (*T*_g_) and crystalline melting point (*T*_m_). It is well known that linear PDMS is a semicrystalline polymer that melts around −40°C, crystallizes at approximately −90°C, and vitrifies upon cooling around −125°C.^22^ The DSC analysis of ionic PDMS melts revealed the expected PDMS *T*_g_ at ≈ −120°C (Figure S16) and a second *T*_g_ attributed to the ionic component of the copolymer, in the range of 70–85°C. The latter assignment is supported by analysis of the highly functionalized PDMS20K-g-NBr copolymer, which showed a single *T*_g_ at 75°C (Figure S16b). In contrast with the aforementioned systems, where no obvious *MW* effects were observed, the introduction of ionic groups at the chain ends alone impacted the thermal property of the PDMS as a function of *MW*. The PDMS5K-d-NBr exhibited a *T*_g_ of −124°C with no melting peak, whereas the PDMS25K-d-NBr and PDMS50K-d-NBr of higher *MW* additionally revealed single melting peaks at −46°C and −43°C, respectively (Figure S17b). This is consistent with observations that, as with other polymers, very low molecular PDMS crystallizes poorly compared to its high molecular weight counterparts.^47^ Crystallization was also observed in the aminopropyl-terminated PDMS25K-d-NH_2_ precursor, which displayed a melting point of −43°C and a *T*_g_ of −124°C (Figure S17). Double melting peaks were observed at −45° and −38°C for PDMS50K-d-NH_2_, consistent with other reports on the complexities of PDMS crystallization behavior.^47,48^ The thermal stability of the synthesized ionic PDMS copolymers was investigated by TGA. The onset mass loss temperature (*T*_onset_) of the ionic-grafted PDMS copolymers was observed at approximately 200°C and is ascribed to the degradation of propylammonium-grafted groups as observed in a previous work,^49^ whereas the cleavage of siloxane backbone is proposed to occur from 400°C. The former corresponded to ca. 11%, 15%, 17%, and 30% of the sample mass, in line with the expected weight fraction of the grafted propyl-trimethylammonium bromide functional groups and consistent with elemental and ^1^H NMR analysis for the PDMS25K-g-NBr, PDMS50K-g-NBr, PDMS6.5K-g-NBr, and PDMS20K-g-NBr copolymers, respectively (Figure S18). The TGA analysis of the propylammonium-terminated PDMS polymers revealed a higher thermal stability than the ionic-grafted PDMS because of the lower concentration of ionic functional groups, whose degradation resulted in only 0.8%, 1.6%, and 5% of weight loss observed from 200°C for the PDMS50K-d-NBr, PDMS25K-d-NBr, and PDMS5K-d-NBr copolymers, respectively, in line with the expected end group content. As in the case of the ionic-grafted PDMS polymers, the cleavage of the siloxane bonds of the PDMS backbone appeared from 400°C (Figure S18).

**Figure 2:**
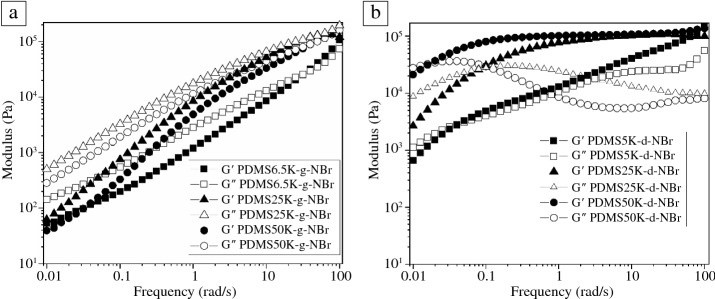
Frequency sweep analysis at room temperature (25°C) of ionic-grafted PDMS (PDMS6.5K-g-NBr, PDMS25K-g-NBr, and PDMS50K-g-NBr) (a), and ionic chain ends of PDMS (PDMS5K-d-NBr, PDMS25K-d-NBr, and PDMS50K-d-NBr) (b).

The viscoelastic properties of the ionic PDMS copolymers were assessed through frequency sweep analysis by measuring the storage and loss moduli *G*′(ɷ) and *G*″(ɷ) as a function of angular frequency at room temperature. The functionalization of ionic interactions increased the viscosity (up to 27 × 10^4^-fold increase for the PDMS5K-d-NBr) compared to their corresponding PDMS precursors. The trimethylammonium-grafted PDMS copolymers (PDMS6.5K-g-NBr, PDMS25K-g-NBr, and PDMS50K-g-NBr) were dominated by a viscous response, with *G*″(ɷ) higher than *G*′(ɷ) over almost the entire range of studied frequencies and with crossover observed only at the highest frequencies (**Figure ****2**a). Similar trends were observed in ionic systems based on phosphonium-functionalized PDMS copolymers with weak ionic interactions.^50^ In contrast to the ionic-grafted PDMS copolymers, the ammonium-terminated PDMS25K-d-NBr and PDMS50K-d-NBr displayed a much greater propensity for solid-like behavior over the range of studied frequencies, with higher plateau moduli versus grafted systems of similar *MW* and with crossover observed at much lower frequencies (i.e., 0.079 Hz and 0.016 Hz, respectively) (Figure 2b). Below the entanglement molecular weight, *M*_*e*_ (12,000 g/mol),^51^ the measured moduli of the PDMS5K-d-NBr dropped and no plateau modulus was observed in contrast with PDMS of higher *MW*, but the material remained solid-like at most frequencies, with a crossover point at 0.039 Hz. One possible explanation for these observations is that repulsion between high concentrations of cationic groups in the grafted systems makes the formation of physical cross-links (in the form of entanglements) exceptionally difficult, thus ensuring that liquid-like behavior dominates in such materials. In contrast, in end-functional systems, the tendency of small concentrations of polar end groups to phase-separate and form clusters with reduced mobility may actually cause the formation of additional physical cross-links versus nonfunctionalized PDMS. This would explain why such a low crossover frequency is observed even in the lowest *MW* end-functionalized PDMS, which would otherwise be expected to behave as a liquid at all frequencies.

### Dispersion of nanosilica in ionic PDMS

Nanosilicas functionalized with sulfonic acid groups were prepared according to previously developed procedures.^28^ The functionalization of the silica surface with 3-(trihydroxysilyl)-1-propane sulfonic acid was confirmed by solid-state ^13^C NMR and ^29^Si NMR analysis as shown in Figures S13 and S14, respectively. DLS analysis of nanosilicas in aqueous solution revealed a unimodal distribution with a diameter of 19 nm and a PDI lower than 0.1 (Figure S15). A sulfur content of 6 ± 1.3 wt% was estimated by elemental analysis of dry sulfonate-functionalized nanosilicas. The thermogram of the sulfonate-functionalized nanosilicas indicated a *T*_g_ of 54°C, which was assigned to the grafted propane sulfonic acid groups. TGA of dry nanosilicas showed 20 wt% mass loss (Figure S19), equivalent to the degradation of propane sulfonic acid groups containing 5.2 wt% sulfur and consistent with the composition obtained by elemental analysis. PDMS–silica nanocomposite films were prepared by mixing sulfonate-functionalized nanosilicas with trimethylammonium-functionalized PDMS. One of the challenges encountered when targeting high nanosilica dispersion levels in PDMS lies in the poor miscibility of hydrophobic PDMS and hydrophilic nanosilica. Whereas PDMS has low solubility in polar solvents, the grafting of ionic groups along the polymer chains was found to enable the modified polymers described here to dissolve in polar aprotic solvents such as DMSO and DMF. In addition, the same solvents were found to be capable of dispersing sulfonate-functionalized nanosilica without significant aggregation. As such, PDMS nanocomposites were prepared by dissolving the synthesized trimethylammonium-grafted PDMS in DMSO. In tandem, an aqueous nanosilica dispersion was diluted with an equal volume of DMSO and then concentrated under low pressure using a rotovap to remove the water. Nanosilicas dispersed in DMSO were then added to the ionic PDMS solution in a Teflon dish and the mixture was heated at 150°C for 2 h, then dried under vacuum (~100 mbar) at 100°C overnight to yield transparent PDMS–silica nanocomposite films. A second set of PDMS–silica nanocomposites was prepared from trimethylammonium-terminated PDMS5K-d-NBr, PDMS25K-d-NBr, and PDMS50K-d-NBr polymers in the presence of sulfonate-functionalized nanosilicas. Due to lower solubility of the trimethylammonium-terminated PDMS in DMSO, other polar organic solvents had to be tested. PDMS5K-d-NBr polymer was found to be soluble in DMF, and thus the corresponding nanocomposites were prepared following the same procedure as for the ionic-grafted PDMS copolymers in DMSO. Nevertheless, the charge density dropped as the *MW* of the ionic end-functionalized PDMS increased, reducing the solubility of the polymer in polar solvents. Therefore, the PDMS25K-d-NBr and PDMS50K-d-NBr samples had to be prepared in toluene. Again, the same procedures were followed, except that, following the addition of the nanosilica dispersion in toluene to the ionic PDMS solution in a Teflon dish, the mixture was heated at 110°C for 2 h instead of 150°C before being dried under vacuum (~100 mbar) at 100°C overnight.

**Figure 3:**
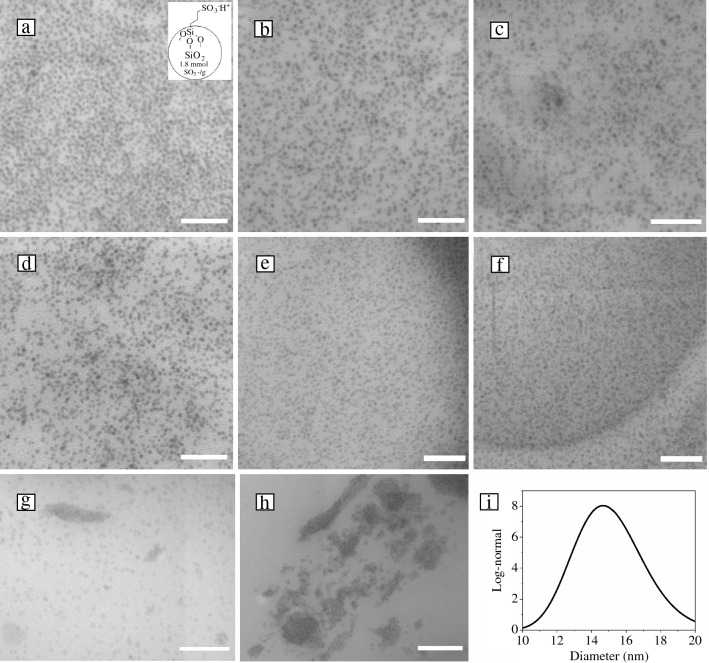
Scanning transmission electron microscope (STEM) images of the bulk from the PDMS–silica nanocomposite films: (a) PDMS20K-g-NBr-Si-10 (inset: nanosilica grafted with sulfonic acid with 1.8 mmol of SO_3_^−^/g); (b) PDMS50K-g-NBr-Si-10; (c) PDMS25K-g-NBr-Si-10; (d) PDMS25K-g-NBr-Si-20; (e) PDMS6.5K-g-NBr-Si-10; (f) PDMS5K-d-NBr-Si-5; (g) PDMS25K-d-NBr-Si-10; (h) PDMS50K-d-NBr-Si-10; and (i) polydispersity fit log-normal distribution of the nanoparticles determined from PDMS20K-g-NBr-Si-10 sample (scale bar 200 nm).

Using the aforementioned approach, a series of PDMS–silica nanocomposite films were prepared by mixing the ionic PDMS with 10 wt% of sulfonate-functionalized nanosilica into different ammonium-functionalized PDMS polymers. The weight fraction of nanosilica in different nanocomposite films was estimated by TGA analysis (Figure S19) and the concentration was found to be between 11 and 14 wt% except for the PDMS20K-g-NBr-10 (20.3 wt%), PDMS5K-d-NBr-10 (16.8 wt%), and PDMS50K-d-NBr-10 (16.9 wt%). This discrepancy may be reflective of an inhomogeneous distribution of nanoparticles in these materials, resulting in higher nanoparticle concentrations in the materials sampled for TGA. To assess nanosilica dispersion levels, scanning transmission electron microscopy (STEM) was performed. The dispersion and distribution of the nanosilica were found to depend on the charge density of the PDMS as well as the corresponding *MW* (**Figure ****3**a–f).

Considering first those systems based on PDMS with ionic group grafted along the chain, well-dispersed nanosilica was observed in all cases, with local modified silica nanoparticle concentrations (as estimated via quantitative image using ImageJ software) for PDMS6.5K-g-NBr (Figure 3e), PDMS25K-g-NBr (Figure 3c–d), and PDMS50K-g-NBr (Figure 3b) corresponding to 15.6 ± 2.0, 17.6 ± 3.7, 21.6 ± 5.0, and 18.1 ± 4.2 wt%, respectively. The observation of high levels of dispersion is consistent with the effects of a combination of repulsive ionic interactions between nanosilica particles and attractive (electrostatic) interactions between nanosilica and the PDMS matrix. The polydispersity fit with log-normal distribution showed a monomodal distribution with a maximum diameter of approximately 15 nm (Figure 3i), which is consistent with DLS analysis. However, the higher-than-expected modified nanosilica content observed via STEM provides evidence of inhomogeneous distribution of nanosilica in the ionic PDMS matrix. Evidence of inhomogeneous nanosilica distribution became more pronounced as the charge density of the ionic PDMS increased, as observed for PDM20K-g-NBr-Si-10 (1.7 mmol/g, **Table** **I**), where particularly high nanosilica loadings (29.5 ± 6.8 wt%) (Figure 3a) were measured locally via STEM image analysis (see also Figure S22a–b). It has been previously reported in ionic polyurethane–silica nanocomposites that optimal dispersion could be reached at a 1:1 ratio between positive and negative charges.^34^ In the ionic PDMS nanocomposites reported here, however, it seems that nanocomposite structure is affected at least as strongly by ionic group content in the polymer chains as by the charge balance of the overall system due to the domination of kinetic effects over thermodynamic effects during nanocomposite preparation. This may be explained by considering the nanocomposite formation process, which involved combining polymer and filler in a common solvent that was subsequently removed through vaporization. Although nanosilica is expected to be both highly mobile and homogenously distributed at the beginning of such a process, its mobility will decrease on solvent removal, enabling the formation of kinetically trapped nanostructures. Indeed, the nanocomposites' preparation method based on solvent casting affects the dispersion of nanoparticles in randomly charged PDMS melts. A molecular dynamics simulation study^52^ demonstrated the impact of weak or strong interactions between nanoparticles and polymer matrix, and the evaporation rate of the solvent on the dispersion state of nanoparticles. A similar behavior was observed by Maguire et al. in ternary polymer nanocomposites.^53^ The overall picture described here is supported by coarse-grained simulations,^35,54^ where nanoparticle dispersion was found to depend on the ratio of electrostatic strength (Bjerrum length: l_B_) and distance between charges rather than the *MW* of the matrix polymer.

**Table I:** Thermal, mechanical, and rheological properties of ionic PDMS and corresponding ionic PDMS–silica nanocomposites.

Polymer	MW (Kg/mol)	Cation Content (mmol/g)	Modified SiO_2_ Content (wt%)		Target Anion-to-Cation Ratio	SiO_2_ Dispersed?	Thermal Transitions (DSC, °C)			Rheological Properties (1 Hz, RT)				Tensile Properties (10 mm/min, *n* = 3)	
			Via* TGA*	Via* STEM* (*n* = *3*)			*T*_g1_	*T*_g2_	*T*_m_	*G*′ (kPa)	*G*″ (kPa)	*η* (kPa·s)	η* (kPa·s)	Break Stress (MPa)	Break Strain (%)
PDMS6.5K-g-NBr	6.5	0.87	–	–	0	Yes	−119	71	–	1.2	2.8	0.5	1.10^–4^	0.16 ± 0.03	118 ± 1
			14.2	15.6 ± 2	0.2	Yes	−121	75	–						
PDMS25K-g-NBr	25	0.69	–	–	0	–	−119	80	–	8.4	17.5	3.1	8.10^–4^	0.41 ± 0.01	99 ± 6
			6.9		0.2	Yes	−122	80	–					0.93 ± 0.14	68 ± 10
			12.2	17.6 ± 3.7	0.3	Yes	−122	81	–					0.34 ± 0.02	35 ± 5
			16.6		0.5	Yes	−122	61	–					0.21 ± 0.07	20 ± 1
			17.1	21.6 ± 5.0	0.7	Yes	−119	71	–						
PDMS20K-g-NBr	20	1.73			0	–	–	76	–				1.10^–3^	0.16 ± 0.01	16 ± 5
			20.3	29.5 ± 6.8	0.1	Yes	–	68	–						
PDMS50K-g-NBr	50	0.83			0	–	−117	85	–	4.8	11.4	1.9	2.10^–3^	0.18 ± 0.05	25 ± 1
			12	18.1 ± 4.2	0.2	Yes	−119	76	–						
PDMS5K-d-NBr	5	0.58			0	–	−124	–	–	13.1	10.6	2.7	1.10^–4^		
			9.6		0.2	Yes	–	67	−45	6.0	1.3	0.9			
			16.8		0.3	Yes	–	72	−45	6.3	1.7	1.0			
PDMS25K-d-NBr	25	0.14			0	–	–	–	−46	77.4	24.3	12.9	1.10^–3^		
			10.6		1.4	No	–	60	−38						
PDMS50K-d-NBr	50	0.07			0	–	–	–	−43	101	8.6	16.1	5.10^–3^		
			16.9		2.9	No	–	–	−37						

*Viscosity of unmodified PDMS melts.

PDMS–silica nanocomposites prepared from PDMS bearing ionic groups at the chain ends were also tested to investigate the impact of charge location on the dispersion of nanosilica. Although the use of PDMS with *MW* above 25,000 g/mol resulted in nanoparticle aggregation (Figure 3g−h), the PDMS5K-d-N-based nanocomposites of higher charge density displayed improved nanosilica dispersion (Figures 3f, S23c). This observation of high levels of dispersion in a non-entangled (ionic) PDMS matrix is in contrast with the observation of no nanosilica dispersion in a non-entangled polystyrene matrix.^55,56^ However, it agrees with a prior report that layered silicates may be dispersed in non-entangled PDMS with polar end groups, and that, more broadly, layered silicate dispersion in PDMS is driven by polar group content.^57^

### Mechanical properties of ionic PDMS–silica nanocomposite

**Figure 4:**
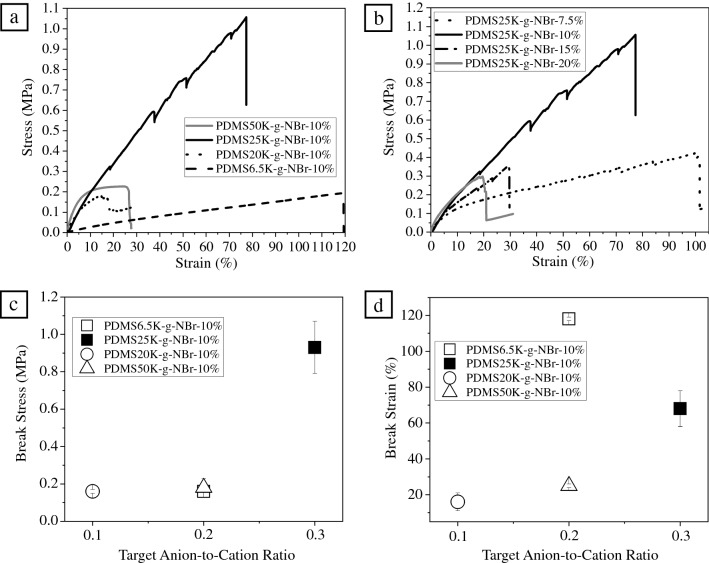
Representative tensile curves for selected poly(dimethylsiloxane)–silica nanocomposites: (a) Effect of the molecular weight and charge density of the ionic PDMS containing 10 wt% of nanosilica; (b) effect of nanoparticle loadings in PDMS25K-g-NBr polymer matrix; (c) effect of anion-to-cation ratio on the break stress of the ionic PDMS nanocomposites containing 10 wt% of nanosilica; (d) effect of anion-to-cation ratio on the break strain of the ionic PDMS nanocomposites containing 10 wt% of nanosilica.

While the unfilled PDMS melts here were too weak/liquid-like in character to enable such work, quasi-static tensile testing was performed on trimethylammonium-grafted PDMS-based nanocomposite films (Figures S20–S21). Tensile test analysis was performed on a PDMS–silica nanocomposite film prepared from PDMS6.5K-g-NBr and 10 wt% of silica. Although rheometry revealed viscous behavior influenced by interactions between cationic groups within the PDMS chains, the addition of nanosilica resulted in mechanical reinforcement with a tensile strength of 0.16 MPa ± 0.03 and a break strain of 118 ± 1% (**Figure ****4**a). Increasing the *MW* of ionic PDMS to 25,000 g/mol (above the entanglement *MW*) resulted in significantly greater mechanical performance at 10 wt% nanosilica loading, with a tensile strength of 0.91 ± 0.20 MPa, but a reduced break strain of 68 ± 12% (Figure 4a). This decrease in break strain was interpreted as indicative of higher nanosilica mobility in a low *MW* matrix.^58,59^

The mechanical reinforcement by nanosilica in a higher *MW* PDMS copolymer of 50,000 g/mol was also tested. PDMS50K-g-NBr containing 10 wt% nanosilica exhibited lower mechanical strength (0.18 MPa ± 0.05) and break strain (25 ± 1%) than the corresponding lower *MW* PDMS25K-g-NBr-based nanocomposites. When considering the tensile performance of nanocomposites based on 10 wt% nanosilica in PDMS with ionic grafts (Figure 4c), it appears that the failure behavior is most strongly influenced by extent of charge balance. This is demonstrated in Figure 4c–d, where we plot break stress and break strain versus target anion-to-cation ratio. The one outlier in this comparison is the exceptionally high break strain observed in the PDMS6.5K-g-NBr-based nanocomposite (Figure 4d). One potential explanation for this may be found in reports of higher nanosilica mobility in a low MW matrix. A PDMS–silica nanocomposite containing 10 wt% of aggregated nanosilica within a cross-linked PDMS matrix has been previously reported with a better mechanical reinforcement (1.8 MPa) than our system due to the covalently cross-linked PDMS matrix.^22^ Lower mechanical reinforcement of approximately 1 MPa was measured for a PDMS–silica nanocomposite where PDMS chains were covalently attached onto silica nanoparticles to improve the dispersion of silica nanoparticles in the PDMS matrix.^26^

Given the levels of mechanical performance observed with 10 wt% nanosilica, the PDMS25K-g-NBr matrix was chosen to study the effects of variations in nanosilica loading (Figure 4b). Decreasing the nanosilica loading to 7.5 wt% gave results in line with the expected reductions in cross-link density, with a lower break stress (0.41 ± 0.01 MPa) and a higher break strain (90 ± 6%) observed. Although better reinforcement might be expected at higher nanoparticle loadings (20 wt%) given an anion-to-cation ratio closer to 1, the onset of nanosilica aggregation resulted in nanoparticles clustering and poor mechanical reinforcement when the nanosilica loading was doubled. A tensile strength of only 0.21 ± 0.07 MPa and a strain at break of 20 ± 1% were measured, confirming the optimum nanosilica loading as 10 wt%. The same effect was observed when the charge density of the polymer was doubled (to 1.7 mmol/g in the case of PDMS20K-g-NBr) while maintaining a nanoparticle loading of 10 wt%, resulting in the lowest observed break stress and break strain values (0.16 ± 0.01 MPa and 16 ± 5%, respectively). Here as well, poor reinforcement arises an inhomogeneous distribution of nanosilica as shown by STEM analysis (Figure S22b). These results further support prior arguments concerning the generation of kinetically trapped nanostructures during ionic nanocomposite formation.

For end-functionalized PDMS5K-d-NBr, mechanical reinforcement was assessed by rheology because no freestanding polymer film could be prepared. In this case, nanosilica addition was observed to reduce the RT storage modulus at both 5 and 10 wt% nanosilica. While a substantial reduction in loss tangent of the nanocomposite versus that of the unfilled polymer was observed in both cases, as would be expected given an increase in cross-link density, the apparent reduction in absolute stiffness was a surprise. One possible explanation for this is that the clustering of the chain ends in the unfilled polymer, previously posited to give rise to its significant increase in solid-like character, might be disrupted through nanosilica addition, with the subsequent interactions with the nanosilica being unable to make up for this change. More study is needed to fully understand this phenomenon, however.

### Self-healing of ionic PDMS nanocomposites

**Figure 5:**
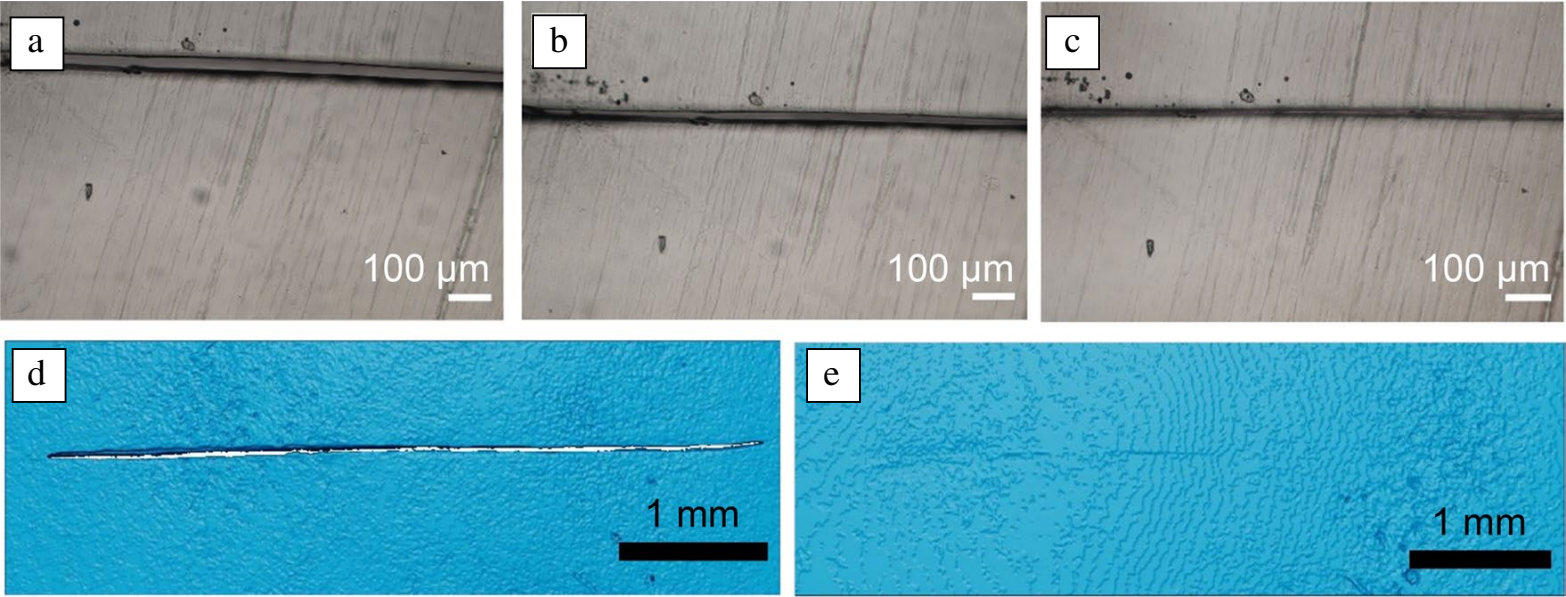
The self-healing behavior of a PDMS6.5K-g-NBr-10% nanocomposite film, with optical microscopy showing the initial scratch (a) before and after thermal treatment (80°C) in a humid environment for (b) 30 min and (c) 1 h. Micro-computed x-ray tomography (micro-CT) results of the same specimen confirm this behavior, showing the entire scratch (d) before healing and (e) after healing for 1 h at 80°C in a humid environment.

Cross-linking PDMS through ionic interactions promises the possibility of self-healing behavior given their reversible nature. This was tested by creating a scratch on the surface of various ionic PDMS nanocomposite films and monitoring the scratch over time, both by optical microscopy and by micro-computed x-ray tomography (micro-CT). Two polymer matrix materials were chosen, namely PDMS25K-g-NBr (which gave a nanocomposite with the highest break stress of any tested) and PDMS6.5K-g-NBr (which gave a nanocomposite with the highest break strain of any tested). The film samples (180 ± 20 µm thick) were placed on a glass substrate and a scratch was created a using a scalpel, giving a scratch width of 25 ± 5 µm (**Figure** **5**a). A healing temperature of 80°C was chosen based on the observation of a glass transition between 70°C and 85°C (Table I) previously assigned to the ionic groups in the system. It was posited that the heating of the ionic nanocomposite films to temperatures close to the glass transition would induce scratch healing as previously demonstrated in polyurethane-based ionic nanocomposites.^34^ The first healing tests were performed for 24 h at 80°C on three samples: PDMS25K-g-NBr-10%, PDMS25K-g-NBr-7.5%, and PDMS6.5K-g-NBr-10 percent. However, no healing was observed in any case (Figure S24a–f). It was hypothesized that the high strength of the ionic interactions between the nanoparticles and the PDMS matrix might be impeding healing under these conditions. To test this, the healing experiment was repeated with the sample (Figure 5a) placed in a humid environment (a sealed glass desiccator with water placed below the sample shelf in place of desiccant; RH = 100%), then heated to 80°C for 24 h. In this case, optical microscopy revealed that, among the three samples, the PDMS6.5K-g-NBr-10% possessed the ability to heal rapidly, presumably due to the higher mobility of nanosilica in a non-entangled PDMS matrix. A decrease in scratch width in this system was observed after 30 min (Figure 5), and after 1 h, complete healing of the scratch was observed (Figure 5c). This was confirmed with micro-CT analysis, which revealed a scratch 5 mm and 25 ± 5 µm wide prior to healing (Figure 5d) versus the near-complete disappearance of the scratch following healing in a warm and humid environment (Figure 5e). These results confirm the sensitivity of the ionic interactions in these systems to the presence of moisture and its very strong impact on nanoparticle mobility and relaxation of the polymer chains. For nanocomposites based on PDMS25K-*g*-NBr, healing was also observed in the case of PDMS25K-*g*-NBr with 7.5 wt% nanosilica (the nanosilica loading giving the highest break strain; Table I, Figure 4b), but with a much longer healing time of 16 h (Figure S22g–h). The longer healing time is arising from reduced polymer chain mobility due to the presence of entanglements in combination with a lower cation content compared to the PDMS6.5K-g-NBr polymer (Table I). Finally, in the case of PDMS25K-g-NBr-10%, no healing was observed over the course of 24 h. Here, it was posited that the higher nanosilica content imposes additional limitations on polymer chain mobility, reducing the healing ability of the nanocomposites. Another possibility to assess the self-healing of materials was to measure the recovery of their mechanical properties after damage and healing. To this end, tensile testing analyses of nanocomposite polymer films were carried out. Applying a scratch along the entire width of the strip and oriented perpendicular to the uniaxial deformation on the surface of the PDMS nanocomposite film reduced its mechanical property as the film broke into two parts at low strain (less than 1 mm) (Figure S25). In parallel, the scratched films were placed in a humid environment then heated to 80°C for 1 h. The tensile testing analysis of the healed PDMS–silica nanocomposites resulted in 73 ± 20% recovery of toughness (Figure S25), thus evidencing the impact of the healing treatment on the recovery of the mechanical property of the nanocomposite films.

### Modeling of cross-links in ionic nanocomposites

Complementing the experimental work described here, we have used coarse-grained molecular dynamics simulations of both ionic and traditional (attractive, nonionic) nanocomposites to shed light on the formation of ionic cross-links between ionically functionalized nanosilicas and PDMS chains. The model is composed of spherical nanoparticles with surface beads, to mimic nanosilicas, and multibead-spring linear polymer chains (Kremer–Grest model) with *N* = 200 monomers (this corresponds to a ratio of *N*/*N*_e_ > 2, where *N*_e_ is the number of monomers between entanglements^60^) at two nanoparticle mass fractions (*w*_p_ = 0.09, *w*_p_ = 0.17). Polymeric and surface beads may or may not carry a permanent charge, whereas the nanocomposite systems are overall neutral in each case. Details about the simulations and the model used in this study can be found elsewhere in the literature.^35^ We model polymers of different charge densities, charged either on their chain ends or along the backbone (one charge every three monomers = p3 type; one charge every 10 monomers = p10 type; one charge every 25 monomers = p25 type), or entirely without charges but incorporating a short-range attraction between polymers and the nanoparticle surface.^61^ It is observed by coarse-grained simulations^35,54^ that nanoparticle dispersion depends on the ratio of electrostatic strength to distance between charges, rather than on the matrix *MW*.

**Figure 6:**
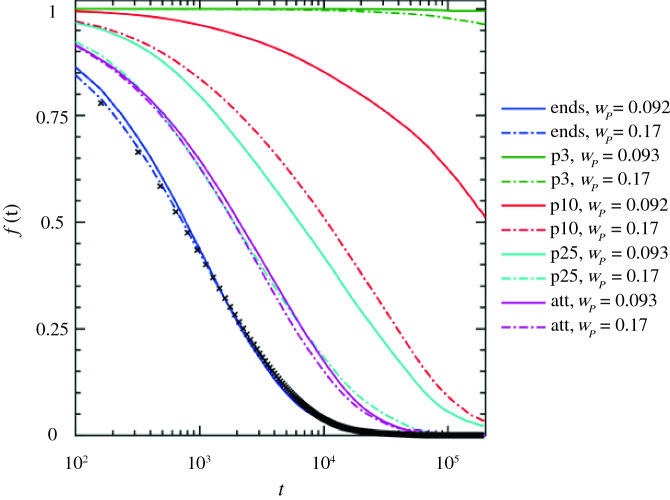
Survival probability, *f*(*t*), that a polymer–nanoparticle cross-link remains intact at *t*, given that it existed at an arbitrary reference time *t* = 0, for various nanoparticle mass fractions (solid lines: *w*_p_ = 0.09, dashed lines: *w*_p_ = 0.17) and polymer types. All the solid lines represent systems where nanoparticles have been dispersed in the matrix (ε_r_ = 24). Black crosses show a nanocomposite with charged ends' polymer matrix (ε_r_ = 48) at *w*_p_ = 0.17 where nanoparticle aggregation is observed.

We define that a temporary cross-link between a polymer chain and a nanoparticle is formed when at least one monomer unit of the polymer chain comes within one atomic diameter of the nanoparticle surface. We showed, in our previous work, that the probability distribution of cross-links, *P*(*X*) (where *X* is the number of different nanoparticles simultaneously involved in cross-links), becomes broader with nanoparticle loading and number of monomer units.^35^ In particular, we calculated the survival probability *f*(*t*) of such a temporary cross-link as a function of time *t*, given that it existed at some reference time *t* = 0. Survival probability of temporary cross-links does not seem to change with the nanoparticle mass fraction at low loadings in the case of nanocomposites based on p3 type polymers, as can be seen in** Figure ****6**, meaning these may be seen as “permanent” cross-links in that case. However, for lower polymer charge densities, such as in p10 type of polymers, *f*(*t*) reduces to 50% of its initial value during the timespan of the simulation.

An increase in nanoparticle loading results in a reduction in *f*(*t*) (except for end-functionalized polymers) because the charges in the polymer chains are distributed over a larger nanoparticle surface area. Likewise, decreases in the polymer charge density lead *f*(t) to decay to 0 more and more rapidly, with the polymer with the lowest charge density (p25 type) giving more or less identical results to the uncharged polymer with simple attractive interactions (att type).^45^ Polymer charge density alters *f*(*t*) substantially, whereas nanoparticle loading has much less of an impact on *f*(*t*) behavior, especially at extreme (low or high) polymer charge densities. This implies that the dynamics of ionic nanocomposites is enhanced when the polymer charge density is decreased, leading to a higher nanoparticle mobility and resulting in temporary ionic cross-links between polymer chains, as speculated in a previous study of ionic polyurethane–nanosilica composites.^34^

Whereas all the simulated ionic nanocomposites with a dielectric constant value ε_r_ = 24 of the matrix (solid lines in Figure 6) lead to nanoparticle dispersion, the end-charged polymer nanocomposite with ε_r_ = 48 (*w*_p_ = 0.17) exhibits nanoparticle aggregation (black crosses in Figure 6). Although the *f*(*t*) behavior of the end-charged polymers is basically unaffected by ε_r_, the increased ε_r_ (smaller Bjerrum length l_B_) leads to a weaker electrostatic strength between ionic polymers and nanoparticles, causing faster translational dynamics of polymers compared to nanoparticles (Figure S26). Thus, our simulations reveal that the degree of ionic nanoparticle dispersion is affected not only by charge sequence and charge density through ionic cross-links,^35^ but also by the electrostatic strength (Bjerrum length: l_B_), which can affect the ratio of polymer to nanoparticle dynamics. In a nanocomposite with dispersed nanoparticles, there is an enhanced layer of monomers at the nanoparticle surface, thus there is an increase in the energy required for a monomer exchange from the 1st to 2nd coordination shell around the nanoparticle.^62^

The time that is needed for the survival probability, *f*(*t*) of ionic cross-links (for the charged ends' polymers) is ~4 × 10^4^ $\uptau$, where $\uptau$ is the Lennard–Jones (LJ) time, given by $\uptau =\sqrt{{{\mathrm{m}\upsigma }_{\mathrm{mp}}}^{2}/\upvarepsilon }$, where *m* is the mass of a monomer atom, ε is the interaction energy, and *σ*_mp_ = (*σ*_m_ + *σ*_p-bead_)/2 is the arithmetic mean distance between a bead of the nanoparticle surface (*σ*_p-bead_) and a monomer (*σ*_m_). By mapping the LJ time to real units (see “Methods” section in Supporting Information), we calculate $\uptau =0.495 \,{\text{ps}}$, which translates to a time for the *f*(*t*) of ionic cross-links with charged ends' polymers equal to ~19.8 ns. The ion pair lifetimes in ionic liquids^63^ and in ionically tethered canopies with opposite ionic nanosilicas^30^ have been measured by PFG-NMR and NMR relaxation and are of the orders of nanoseconds (*ns*), which are in agreement to the time measurement by simulations in this work.

## Conclusions

A library of PDMS-based polymers containing cationic groups was successfully synthesized and characterized to study the impact of charge density and charge location on the structure and properties of nanocomposites formed with oppositely charged nanosilica. Thermal analysis of ionic polymers revealed higher thermal stability in the case of end-functionalized PDMS versus cation-grafted PDMS. The viscoelastic properties of these materials as assessed by frequency sweep experiments in a parallel plate rheometer showed evidence of a significant viscous response in the case of cation-grafted PDMS, whereas end-functionalized PDMS exhibited solid-like behavior. The dispersion and distribution of nanosilica in these ionic PDMS matrix materials were driven at least as much by charge location and charge density as by charge balance, with good dispersion possible in all cation-grafted PDMS (albeit with more and more inhomogeneous nanosilica distribution observed as charge density increased), whereas in the end-functionalized PDMS case, only the system with the highest charge density produced high levels of nanosilica dispersion. The highest break stress was observed in the entangled PDMS25K-g-NBr (*MW* > *M*_e_) with a nanosilica loading of 10 wt%. Here, the relatively low overall charge density helps to avoid the formation of a kinetically trapped nanostructure exhibiting inhomogeneous nanosilica distribution. High levels of nanoscale dispersion coupled with uniform distribution and an entangled polymer phase all contribute to increased break stress. The highest break strain was observed in PDMS6.5K-g-NBr with a nanosilica loading of 10 wt%. This is ascribed to the higher mobility of nanosilica dictated thanks to the presence of non-entangled PDMS chains. The aforementioned nanocomposite also displays rapid (within 30–60 min) self-healing at 80°C in a humid environment, consistent with the proposition of high mobility coupled with the reversible nature of ionic interactions. Finally, coarse-grained simulations show that the lifetime of ionic cross-links depends strongly on the polymer charge density, implying that charge density should also affect nanoparticle mobility in the polymer matrix.

## Materials and methods

### Materials

(20–25% aminopropylmethylsiloxane)-*r*-dimethylsiloxane copolymer 900–1100 cSt (AMS-1203); (6–7% aminopropylmethylsiloxane)-*r*-dimethylsiloxane copolymer 1800–2200 cSt (AMS-163), aminopropyl-terminated polydimethysiloxane 100–120 cSt (DMS-A21), aminopropyl-terminated polydimethysiloxane 900–1100 cSt (DMS-A31); aminopropyl-terminated polydimethysiloxane 4000–6000 cSt (DMS-A35), (7–9% methylhydrosiloxane)-*r*-dimethylsiloxane copolymer trimethylsiloxane-terminated 110–150 cSt (HMS-082), and (4–6% methylhydrosiloxane)-*r*-dimethylsiloxane 750–1000 cSt (HMS-053) were purchased from Gelest; bromomethane was purchased from ABCR; Ludox HS-30 colloidal silica, 3-(trihydroxysilyl)-1-propane sulfonic acid, *N,N*-dimethylallylamine, sodium bicarbonate (NaHCO_3_), Platinum(IV) oxide, organic solvents (purity > 95%), and Dowex 50 W X8 highly acidic ion-exchange resin (H + form) were purchased from Sigma-Aldrich; finally, SnakeSkin™ Dialysis Tubing, 10 K MWCO, 22 mm, was purchased from Thermo Fisher.

### Methods

A detailed description of the synthesis of trimethylammonium-functionalized PDMS and the preparation of PDMS–silica nanocomposites can be found in the Supporting Information.

Detailed information concerning characterization can also be found in the Supporting Information, including ^1^H NMR and ^13^C NMR, elemental analysis, differential scanning calorimeter (DSC), thermogravimetric analysis (TGA), tensile test, rheological test, dynamic light scattering (DLS), scanning transmission electron microscopy (STEM), and micro-computed tomography (micro-CT).

## Supplementary information

Below is the link to the electronic supplementary material.

Supplementary file1 (PDF 2028 KB)
