# Supplementary material for: Ionic poly(dimethylsiloxane)–silica nanocomposites: Dispersion and self-healing
Source: MRS Bull. 2022 Sep 16;47(12):1185–97. doi: 10.1557/s43577-022-00346-x (PMC9947054; doi:10.1557/s43577-022-00346-x)
Supplement: Supplementary file 1 — Supplementary file1 (PDF 2028 KB) [file 43577_2022_346_MOESM1_ESM.pdf]

Supporting Information

Ionic Polydimethylsiloxane-Silica Nanocomposites: Dispersion and Self-Healing

Clément Mugemana[1], Ahmad Moghimikheirabadi[2], Didier Arl[1], Frédéric Addiego[1], Daniel F. Schmidt[1], Martin Kröger[2] and Argyrios V. Karatrantos[1]*

[1] Materials Research and Technology, Luxembourg Institute of Science and Technology, 5, Avenue des Hauts-Fourneaux, L-4362 Esch-sur-Alzette, Luxembourg

[2] Polymer Physics, Department of Materials, ETH Zürich, Leopold-Ruzicka-Weg 4, CH-8093 Zürich, Switzerland

Materials

(20-25% aminopropylmethylsiloxane)-dimethylsiloxane copolymer 900-1,100 cSt (AMS-1203); (6-7% aminopropylmethylsiloxane)-dimethylsiloxane copolymer 1,800-2,200 cSt (AMS-163), aminopropyl terminated polydimethysiloxane 100-120 cSt (DMS-A21), aminopropyl terminated polydimethysiloxane 900-1,100 cSt (DMS-A31); aminopropyl terminated polydimethysiloxane 4,000-6,000 cSt (DMS-A35), (7-9% methylhydrosiloxane)-dimethylsiloxane copolymer trimethylsiloxane terminated 110-150 cSt (HMS-082) and (4-6% methylhydrosiloxane)-dimethylsiloxane 750-1,000 cSt (HMS-053) were purchased from Gelest (as commercial polysiloxanes generally possess low PDIs, all polymers used in this work may be considered effectively monodisperse); bromomethane was purchased from ABCR; Ludox HS-30 colloidal silica, 3-(trihydroxysilyl)-1-propane sulfonic acid, *N,N*-dimethylallylamine, sodium bicarbonate ($NaHCO_3$), Platinum(IV) oxide, organic solvents (purity > 95%) and Dowex 50W X8 highly acidic ion-exchange resin (H^+ form) were purchased from Sigma Aldrich; finally, SnakeSkin™ Dialysis Tubing, 10K MWCO, 22 mm was purchased from Thermo Fisher.

Methods

NMR spectra were recorded on a Bruker AVANCE III HD spectrometer (600 MHz) at 298 K in deuterated chloroform which was used as an internal standard for ^{1}H and ^{13}C NMR. Thermogravimetric analysis (TGA) was carried out under N_2 on a TGA2 STARe System (Mettler Toledo, Switzerland) applying a heating rate of 10 °C min^{-1}. DSC traces were recorded on a DSC 240 F1 system (Netzsch, Germany), with samples heated and cooled at 20 K/min and analyzed over a temperture range of -170°C to 200°C in flowing nitrogen. The data were collected and analyzed using Proteus Thermal Analysis software (Netzsch, Germany). Glass transition temperatures were determined based on the midpoint of the transition for the second heating cycle. Elemental analysis (CHNS) measurements were performed on a Vario MACRO cube CHNS/O analyzer from Elementar France SARL, operating based on the Pregl-Dumas method. A Leica EM FC6 cryoultramicrotome was used for the cryosectioning of the ionic PDMS nanocomposites at -120°C to yield 50-70 nm thick samples. Morphology and thickness of the obtained samples were characterized using a FEI Helios NanoLab 650 Focused Ion Beam

Scanning Electron Microscope (FIB-SEM). Thin lamellae were analyzed using a STEM detector in bright and dark field modes with an accelerating voltage of 30 kV. The loadings of nanosilica from STEM images analysis were estimated using ImageJ software by counting nanoparticles in a specific area for a thin lamella of 50 nm and 70 nm thick. Frequency sweep measurements were performed using an Anton Paar Physica MCR 302 rheometer equipped with a CTD 450 temperature control device with a disposable aluminum plate–plate (diameter: 15 mm, measure gap: 0.5 mm) geometry. A frequency sweep between 0.01 and 100 Hz was measured with a shear strain amplitude of 1%. The 3D imaging of the sample before and after healing was conducted using an EasyTom 160 a micro-computed x-ray tomograph (μCT) from RX Solutions (Chavanod, France). In particular, attention was focused on the evolution of the scratch morphology during the thermal healing procedure. The sample was positioned on a rigid cardboard using double-sided tape, providing stable mounting during x-ray emission. 2D slices were recorded with a tube voltage of 60 kV and current of 160 μA applied to a tungsten filament. The Source-to-Object Distance (SOD) and the Source-to-Detector Distance (SDD) were set to ~12 mm and ~370 mm, respectively, enabling a voxel size of ~4 μm. A 360° rotation was applied to the sample with steps of 0.25°, resulting in the recording of 1440 slices. The volume reconstruction was conducted utilizing the XAct64 software package (RX Solutions, Chavanod, France) which corrected for sample and spot movement and ring artifacts and reconstructed the volume of interest in a given spatial orientation based on the corrected slices. Subsequent image analysis and 3D visualization were performed using the Avizo software package (ThermoFisher, Waltham, USA). A median filter was first applied as a smoothing treatment to attenuate the noise and soften the edges of the objects. The image contrast was consequently reduced, but its intensity segmentation was significantly facilitated. This filter was applied in 3D using a connectivity of 6 voxels (with a common face with the voxel to be corrected). Then, the intensity was segmented to extract the sample from the background (surrounding air) and the tape. It should be noted, however, that the segmentation between the sample and the tape was not optimal due to a similar nature of the materials. Lastly, the segmented image was visualized in 3D by means of a volume rendering module using cubic interpolation.

Mapping of Lennard Jones time (τ) to real time units

The Lennard-Jones (LJ) time, given by $\tau = \sqrt{m\sigma_{mp}{}^2/\varepsilon}$, where m is the mass of a monomer atom, ε is the interaction energy, and $\sigma_{mp} = (\sigma_m + \sigma_{p\text{-bead}})/2$, is the arithmetic mean distance between a bead of the nanoparticle surface ($\sigma_{p\text{-bead}}$) and a monomer (σ_m). In order to map to real units, we use: mass of a monomer atom, m=$1.660538921 \cdot 10^{-27}$ Kg (1/12 the mass of a ^{12}C atom), interaction energy ε= 2 KJ/mol (since $\varepsilon = k_BT/T^*$, k_BT=2.5 KJ/mol at 300 K, the reduced temperature T*=1.25 is used in the coarse grained simulations), 1 mol=$6.023 \cdot 10^{23}$ atoms, σ_{mp}=(1+0.4)/2=0.7 nm. Substituting the values to LJ time, **τ,** we calculate $\tau = 0.495$ ps

A. **Synthesis of ionic polydimethylsiloxane polymers**

1. Synthesis of poly((*N*,*N*-dimethyl-3-aminopropyl)methylsiloxane)-*co*-dimethylsiloxane) copolymers (PDMS6.5K-*g*-NMe)

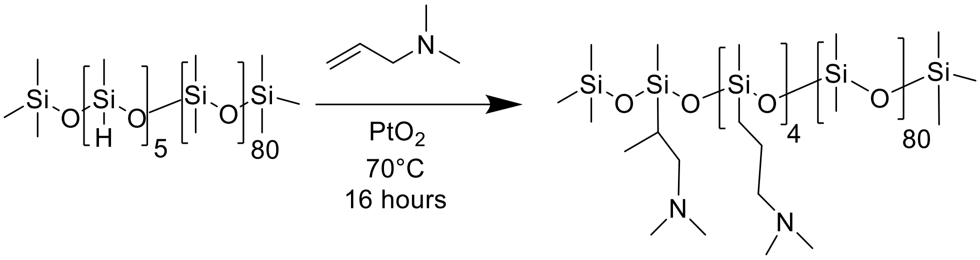

Figure S1. Synthesis of poly((*N*,*N*-dimethyl-3-aminopropyl)methylsiloxane)-*r*-dimethylsiloxane).

The (7-9% methylhydrosiloxane)-dimethylsiloxane trimethylsiloxane terminated copolymer (3.00 g, 0.461 mmoles, 2.83 mmoles of Si-H, 1 eq.) was placed in a Schlenk tube and dried under vacuum at 80°C, overnight. *N*,*N*-dimethylallylamine (0.725 g, 8.513 mmoles, 3 eq.) was added to the Schlenk tube under argon. The PtO_2 catalyst (0.032 g, 0.141 mmoles, 0.05 eq.) was added to the reactant mixture, and the Schlenk tube was placed in an oil bath heated at 70°C under argon, overnight. After the reaction, the viscous brownish solution was diluted in dichloromethane and purified by centrifugation to remove agglomerated PtO_2 catalyst (10.000 rpm, 0°C, 1 hour). The filtered solution was concentrated and dried on a rotovap to yield a clear and colorless viscous oil. Yield (85 %) ^{1}H NMR (600 MHz, $CDCl_3$): δ_H 0-0.2 ppm (s, 512 H, a); 0.5 ppm (m, 7.6 H, b); 0.9 ppm (m, 0.7 H, b'); 1.1 ppm (m, 3.5 H, c'); 1.6 ppm (, 7.4 H, c); 2.3-2.4 ppm (s and m, 40.2 H, d+e); ^{13}C NMR: δ_C 1.2 ppm (**C**H_3-SiO, a); 12.0 ppm (-**C**H_3-CH, c'); 15.0 ppm (-**C**H_2-SiO, b); 19.8 ppm (-**C**H-SiO, b'); 20.7 ppm (-**C**H_2-CH_2, c); 45.1 ppm (-**C**H_3-N, e, e'); 61.5 ppm (CH-**C**H_2-N, d'); 62.7 ppm (-CH_2-**C**H_2-N, d).

2. Synthesis of poly((trimethyl-3-(methylsiloxane)propylammonium bromide)-*r*-dimethylsiloxane) copolymers (PDMS6.5K-*g*-NBr)

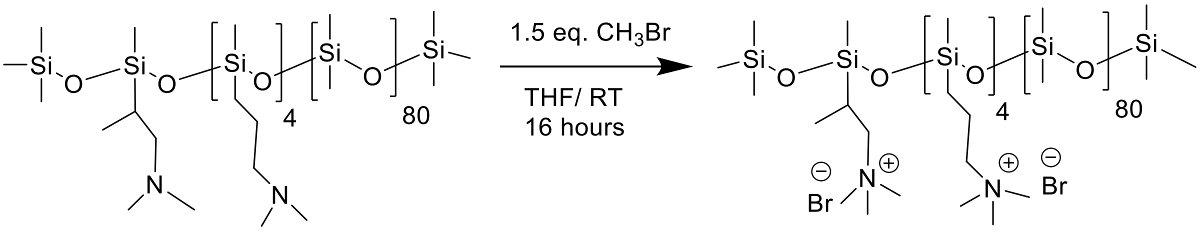

Figure S2. Synthesis of poly((trimethyl-3-(methylsiloxane)propylammonium bromide)-*r*-dimethylsiloxane.

Bromomethane (0.33 g, 3.46 mmol, 1.5 eq.) was transferred from a gas cylinder into a tightly sealed nitrogen tube immersed in liquid nitrogen. In parallel, the PDMS6.5K-g-NMe copolymer (3.0 g, 0.46 mmol, 2.30 mmol of dimethylaminopropyl, 1 eq.) was dissolved in 15 mL of THF and placed in a thick-walled nitrogen tube. Bromomethane was transferred to the solution of the PDMS6.5K-g-NMe copolymer and the reaction mixture was heated at 50°C for 24 hours in a sealed nitrogen tube. After the reaction, the solvent and the excess of methyl bromide were removed under vacuum to yield ionic PDMS6.5K-g-NBr copolymer functionalized with trimethylammonium groups. (Yield: 75%) ^{1}H NMR (600 MHz, $CDCl_3$):

δ_H 0-0.4 ppm (s, 512 H, a); 0.5 ppm (m, 7.6 H, b); 1.3 ppm (m, 1.3 H, b'); 1.4 ppm (m, 3.3 H, c'); 1.7 ppm (t, 7.4 H, c); 3.3-3.7 ppm (s and m, 67.3 H, d+e); ^{13}C NMR: δ_C 1.2 ppm (**C**H_3-SiO, a); 13.5 ppm (-**C**H_2-SiO, b); 15.1 ppm (-**C**H-SiO, b'); 17.2 ppm (-**C**H_2-CH_2, c); 30.5 ppm (-**C**H_2-CH, c'); 53.7 ppm (-**C**H_3-N-Br, e, e'); 69.6 ppm (**C**H_2-N-Br, d).

3. Synthesis of poly((trimethyl-3-(methylsiloxane)propylammonium bromide)-*r*-dimethylsiloxane) copolymer (PDMS20K-g-NBr)

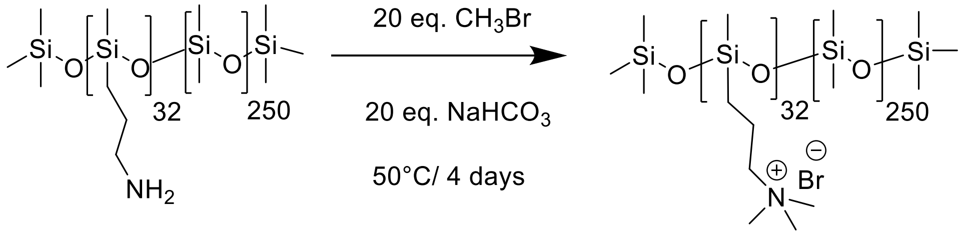

Figure S3. Synthesis of poly((trimethyl-3-(methylsiloxane)propylammonium bromide)-*co*-dimethylsiloxane)

Bromomethane (12.829 g, 135.1 mmol, 20 eq.) was transferred into a tightly sealed nitrogen tube immersed in liquid nitrogen. In parallel, the PDMS20K-g-NH_2 copolymer (2.0 g, 0.15 mmol, 6.75 mmol of aminopropyl, 1 eq.) was dissolved in 20 mL of THF and transferred in a thick-walled nitrogen tube, and mixed with sodium bicarbonate (11.35 g, 135.1 mmol, 20 eq.). Bromomethane was transferred to the solution of PDMS20K-g-NH_2 copolymer, and the reaction mixture was heated at 50°C for 4 days. After the reaction, the ionic PDMS solution was filtered to remove the excess of sodium bicarbonate, and the remaining $NaHCO_3$ salt soluble in THF was extracted by centrifugation (10.000 rpm, 0°C, 1 hour). The filtered solution was dried under vacuum to yield the PDMS20K-g-NBr copolymer functionalized with trimethylammonium groups. ^{1}H NMR (600 MHz, $CDCl_3$): δ_H 0-0.4 ppm (s, 1600H, CH_3-SiO); 0.5 ppm (t, 63H, CH_2-Si); 1.78 ppm (t, 59H, **C**$\mathbf{H_2}$-CH_2-Si); 2.3 ppm (H_2O); 3.4-3.5 ppm (s and t, 351H, $(CH_3)_3$-N^+-CH_2); ^{13}C NMR: : δ_C 1.3 ppm (**C**H_3-SiO, a); 13.7 ppm (-**C**H_2-SiO, b); 17.3 ppm (-**C**H_2-CH_2, c); 53.6 ppm (-**C**H_3-N-Br, e); 69.4 ppm (**C**H_2-N-Br, d).

4. Synthesis of propyl trimethylammonium bromide-terminated polydimethylsiloxane (PDMS5K-d-NBr)

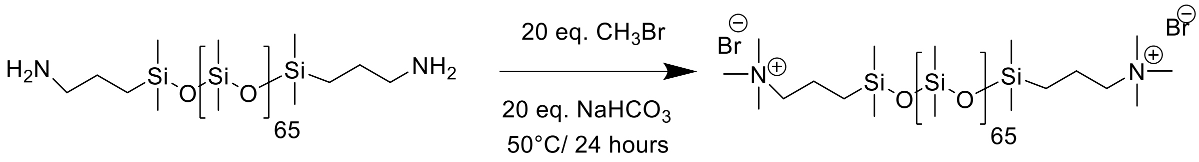

Figure S4. Synthesis of propyl trimethylammonium bromide-terminated polydimethylsiloxane

Bromomethane (2.278 g, 0.024 mol, 20 eq.) was transferred into a tightly sealed nitrogen tube immersed in liquid nitrogen. In parallel, the PDMS5K-d-NH_2 (3.0 g, 0.6 mmol, 1.2 mmol of aminopropyl, 1 eq.) was dissolved in 15 mL of THF and transferred in a thick-walled nitrogen tube, and mixed with sodium bicarbonate (2.016 g, 0.024 mol, 20 eq.). Bromomethane was then transferred to the tightly closed thick-walled nitrogen tube immersed in liquid nitrogen and the reaction mixture was heated at 50°C for 24 hours. After the reaction, the ionic PDMS

solution was filtered to remove the excess of sodium bicarbonate, and the remaining $NaHCO_3$ salt soluble in THF was extracted by centrifugation (10.000 rpm, 0°C, 1 hour). The filtered solution was then dried under vacuum to yield the PDMS5K-d-NBr functionalized with trimethylammonium groups at the polymer chain-ends. ^{1}H NMR (600 MHz, $CDCl_3$): δ_H 0-0.2 ppm (s, 400 H, CH_3-SiO); 0.6 ppm (t, 3.9H, CH_2-Si); 1.8 ppm (t, 3.9H, **CH_2**-CH_2-Si); 2.2 ppm (H_2O); 3.4 ppm (s , 17.8H, (**CH_3**)$_3$-N^+-CH_2); 3.5 ppm (m , 4H, $(CH_3)_3$-CN^+-**CH_2**). ^{13}C NMR δ_C 1.2 ppm (**C**H_3-SiO, a); 14.5 ppm (-**C**H_2-SiO, b); 17.6 ppm (-**C**H_2-CH_2, c); 53.7 ppm (**CH_3**)$_3$-CN^+-CH_2); 69.9 ppm $(CH_3)_3$-CN^+-**CH_2**).

B. Synthesis of ionic PDMS-Silica nanocomposites

1. Surface modification of Silica nanoparticles

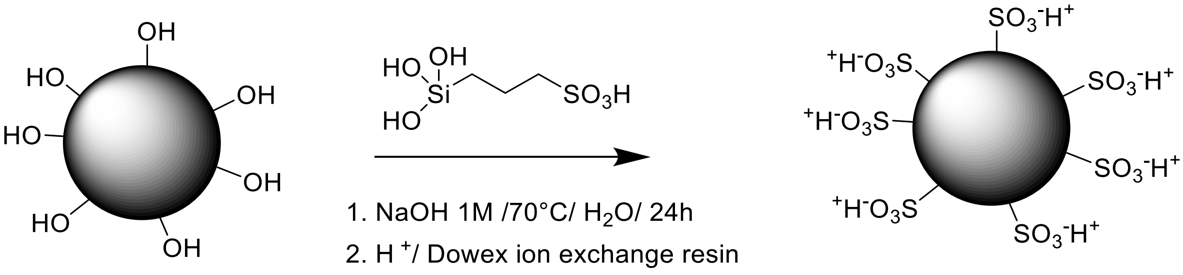

Figure S5. Synthesis of charged silica nanoparticles functionalized with sulfonates groups.

In a two-neck round-bottom 4 g of 3-(trihydroxysilyl)-1-propanesulfonic acid was diluted with 20 mL of deionized water. On the other hand, 3 g of Ludox HS 30 colloidal silica were diluted with 22 mL of deionized water (22 mL). The colloidal silica suspension was slowly added to the 3-(trihydroxysilyl)-1-propanesulfonic acid suspension, while stirring vigorously. To the mixture, a solution of sodium hydroxide solution (1 M) was added dropwise until a pH ~5 was reached. The solution was then heated to 70 °C and stirred vigorously for 24 h. The suspension was then cooled to room temperature, transferred into a dialysis tubing (SnakeSkin™ Dialysis Tubing, 10K MWCO, 22 mm) and dialyzed against deionized water for 3 days while changing water twice a day. The opalescent silica solution was run through an ion exchange column (Dowex 50W X8 ion exchange resin) to exchange Na^+ ions against protons. (d: 19 nm ± 0.1, Sulfur: 6.0 ± 1.3 wt.%).

2. Silica-PDMS nanocomposites

- The synthesis of ionic PDMS-silica nanocomposites from trimethylammonium-grafted polydimethylsiloxane copolymers (PDMS-g-NBr). Example from the PDMS25K-g-NBr-Si-10%. 900 mg of PDMS25K-g-NBr were dissolved in 10 mL of DMSO and transferred in a Teflon PTFE dish of 60 mm of diameter. In tandem, 10 mL of a dispersion of silica nanoparticles (10 g/L) were transferred into round-bottom flask to which 10 mL of DMSO were added. The solvent mixture was concentrated on a rotary evaporator under high vacuum (20 mbar / 60°C) to remove the aqueous phase. The concentrated dispersion of silica nanoparticles in DMSO (10 mL) was added to the solution of PDMS25K-g-NBr (in Teflon dish) and the mixture was heated at 150°C for 2 hours to evaporate the DMSO solvent. The resulting transparent PDMS-silica nanocomposites film was then dried under vacuum (100 mbar) at 100°C overnight. The same process was used for the PDMS6.5K-g-NBr, PDMS20K-g-NBr and PDMS50K-g-NBr copolymers.

- The synthesis of ionic PDMS-silica nanocomposites from trimethylammonium-terminated polydimethylsiloxane (PDMS5K-d-NBr-Si-10%). 900 mg of PDMS5K-g-NBr were dissolved in 10 mL of DMF and transferred in a Teflon PTFE dish of 60 mm of diameter. In tandem, 10 mL of a dispersion of silica nanoparticles (10 g/L) were transferred into round-bottom flask to which 10 mL of DMF were added. The solvent mixture was concentrated on a rotary evaporator under high vacuum (20 mbar / 60°C) to remove the aqueous phase. The concentrated dispersion of silica nanoparticles in DMF (10 mL) was added to the solution of PDMS5K-g-NBr (in Teflon dish) and the mixture was heated at 150°C for 2 hours to evaporate DMF solvent. The transparent PDMS-silica nanocomposites film was then dried under vacuum (100 mbar) at 100°C overnight. For the PDMS25K-d-NBr and PDMS50K-d-NBr, toluene solvent was used instead of DMF due to the low solubility of these polymers in DMF.
- The synthesis of ionic PDMS-silica nanocomposites from trimethylammonium-terminated polydimethylsiloxane (PDMS25K-d-NBr or PDMS50K-d-NBr). 900 mg of PDMS25K-g-NBr were dissolved in 10 mL of toluene and transferred in a Teflon PTFE dish of 60 mm of diameter. In tandem, 10 mL of a dispersion of silica nanoparticles (10 g/L) were transferred into round-bottom flask to which 20 mL of toluene were added. The solvent mixture was concentrated on a rotary evaporator (100 mbar / 40°C) to remove the aqueous phase. The concentrated dispersion of silica nanoparticles in toluene (10 mL) was added to the solution of PDMS25K-g-NBr (in Teflon dish) and the mixture was heated at 110°C for 2 hours to evaporate toluene solvent.

C. Characterization of ionic PDMS copolymers

1. ^{1}H NMR Spectra

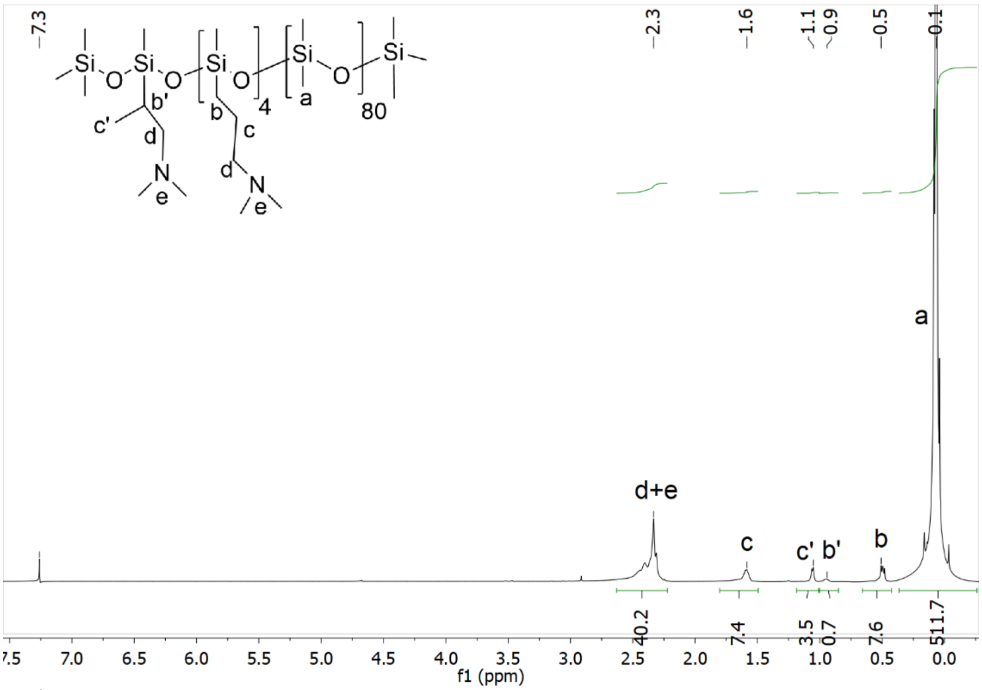

Figure S6. ^{1}H NMR spectrum of the poly((*N*,*N*-dimethyl-3-aminopropyl)methylsiloxane)-*r*-dimethylsiloxane) (PDMS6.5K-g-NMe)

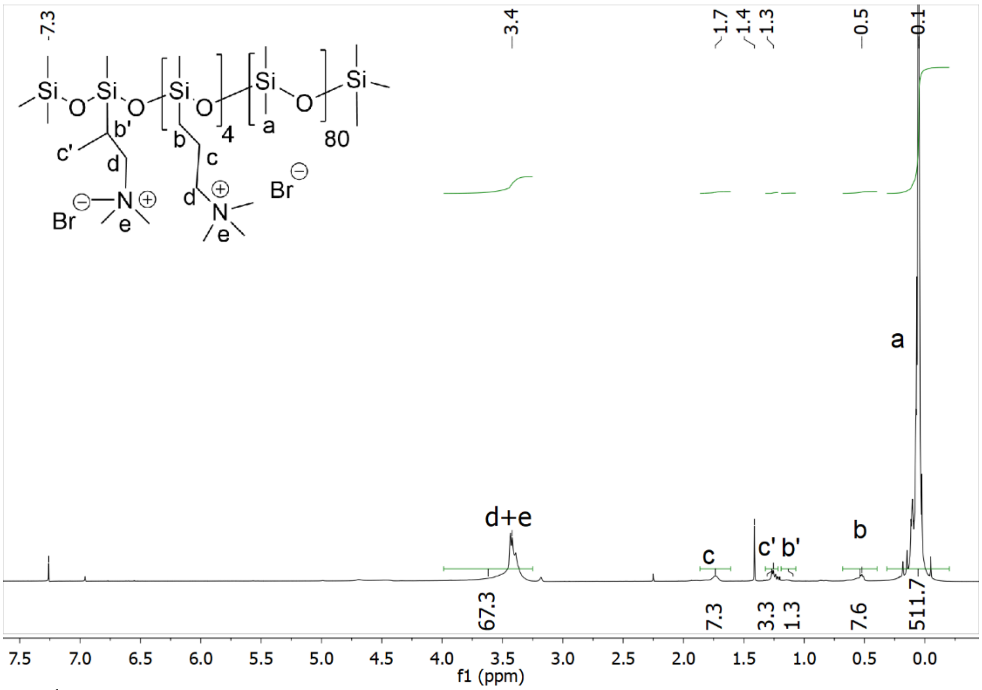

Figure S7. ^{1}H NMR spectrum of the poly((trimethyl-3-(methylsiloxane)propylammonium bromide)-*r*-dimethylsiloxane) (PDMS6.5K-g-NBr)

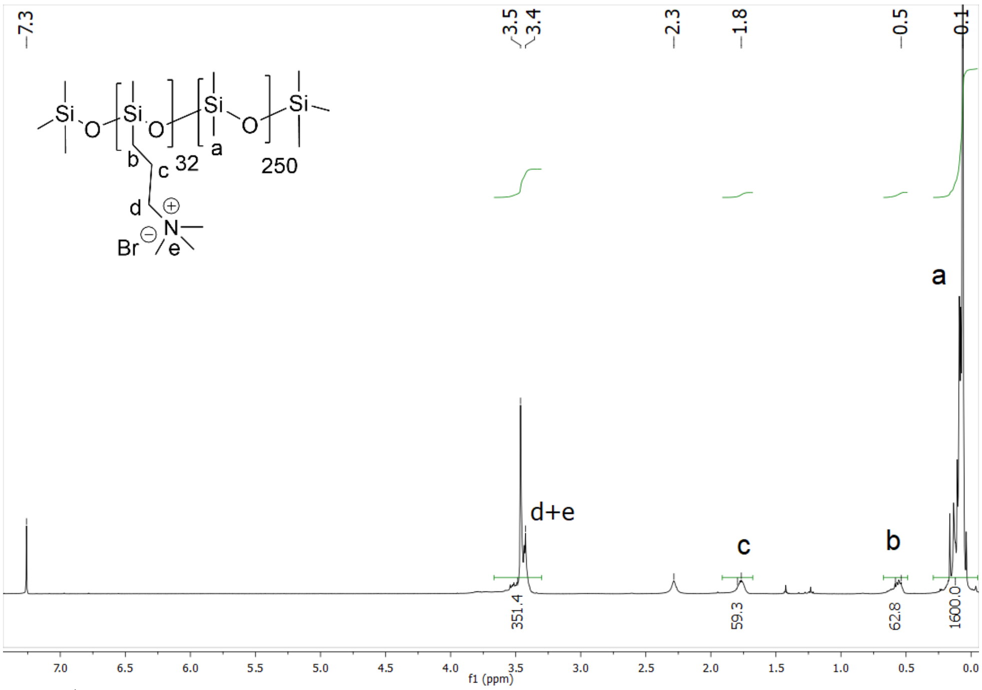

Figure S8. ^{1}H NMR spectrum of poly((trimethyl-3-(methylsiloxane)propylammonium bromide)-*r*-dimethylsiloxane) (PDMS20K-g-NBr)

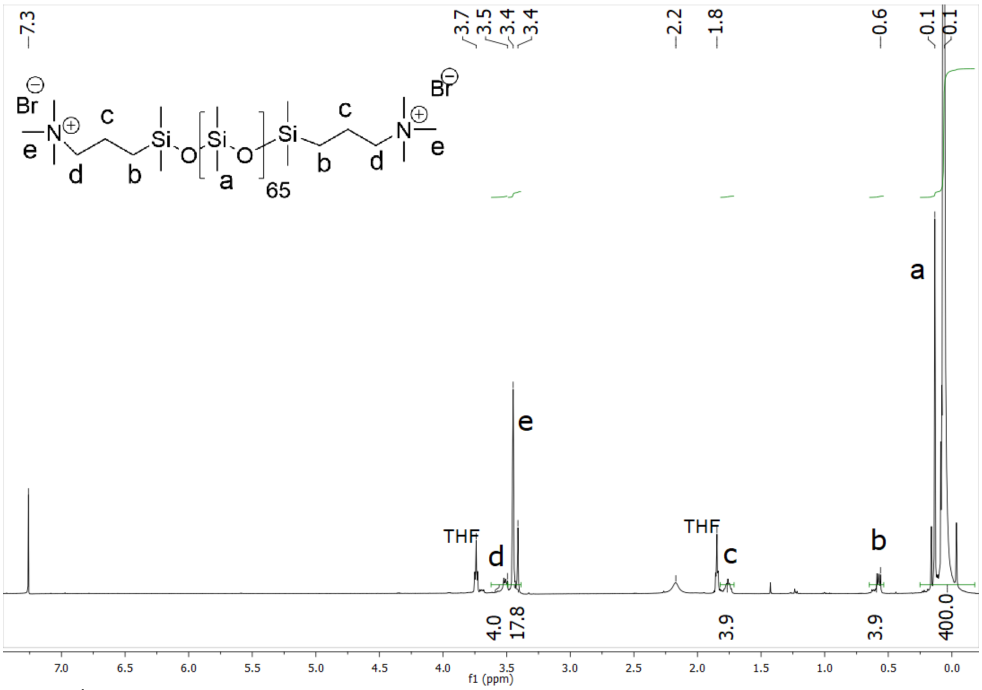

Figure S9. ^{1}H NMR spectrum of propyl trimethylammonium bromide-terminated polydimethylsiloxane (PDMS5K-d-NBr)

2. ^{13}C NMR

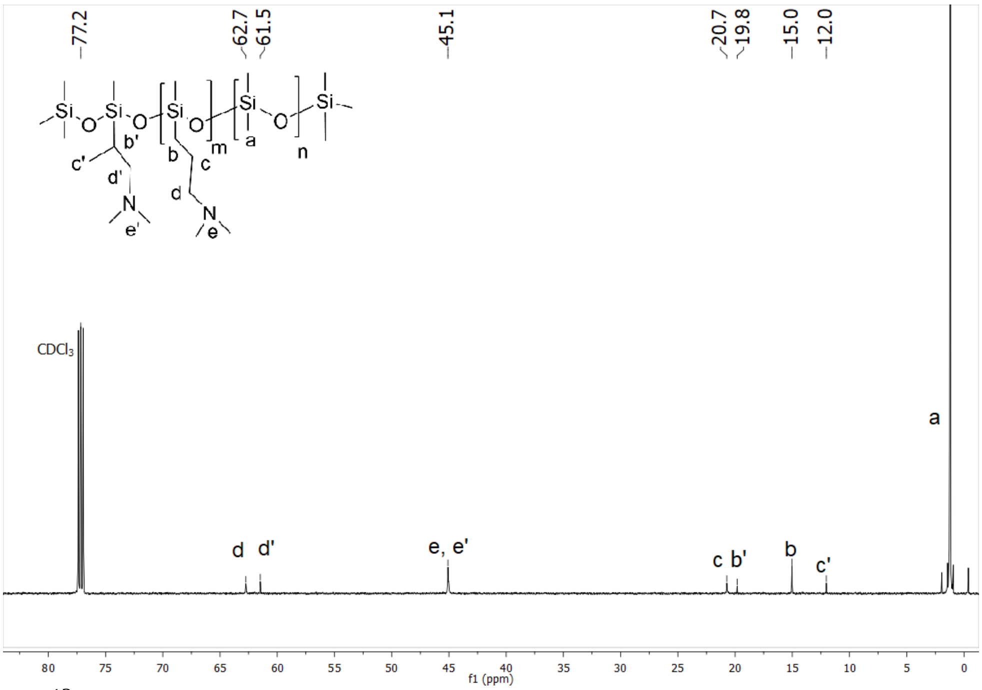

Figure S10. ^{13}C NMR spectrum of the poly((*N*,*N*-dimethyl-3-aminopropyl)methylsiloxane)-*r*-dimethylsiloxane) (PDMS25K-g-NMe)

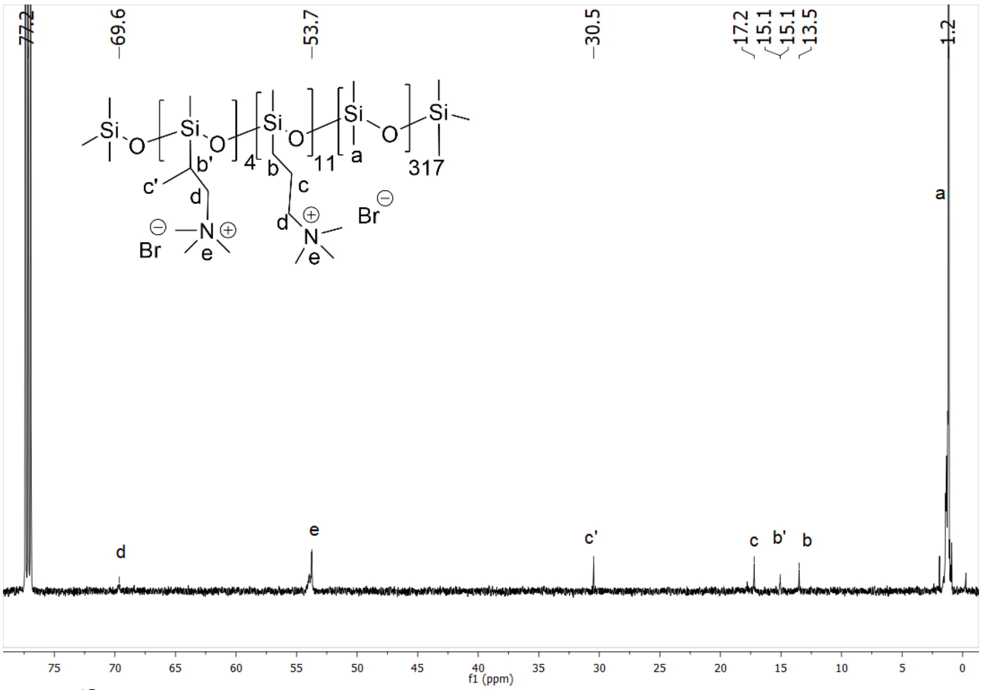

Figure S11. ^{13}C NMR spectrum of poly((trimethyl-3-(methylsiloxane)propylammonium bromide)-*r*-dimethylsiloxane) (PDMS25K-g-NBr)

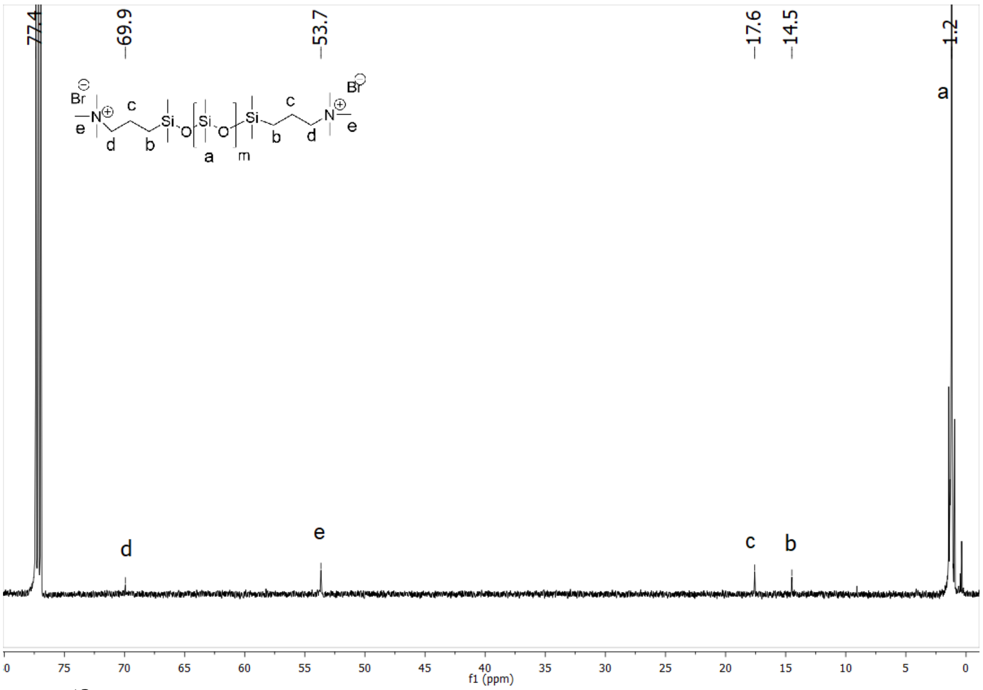

Figure S12. ^{13}C NMR spectrum of the propyl trimethylammonium bromide-terminated polydimethylsiloxane (PDMS5K-d-NBr)

D. Characterization of ionic functionalized silica and PDMS-silica nanocomposites

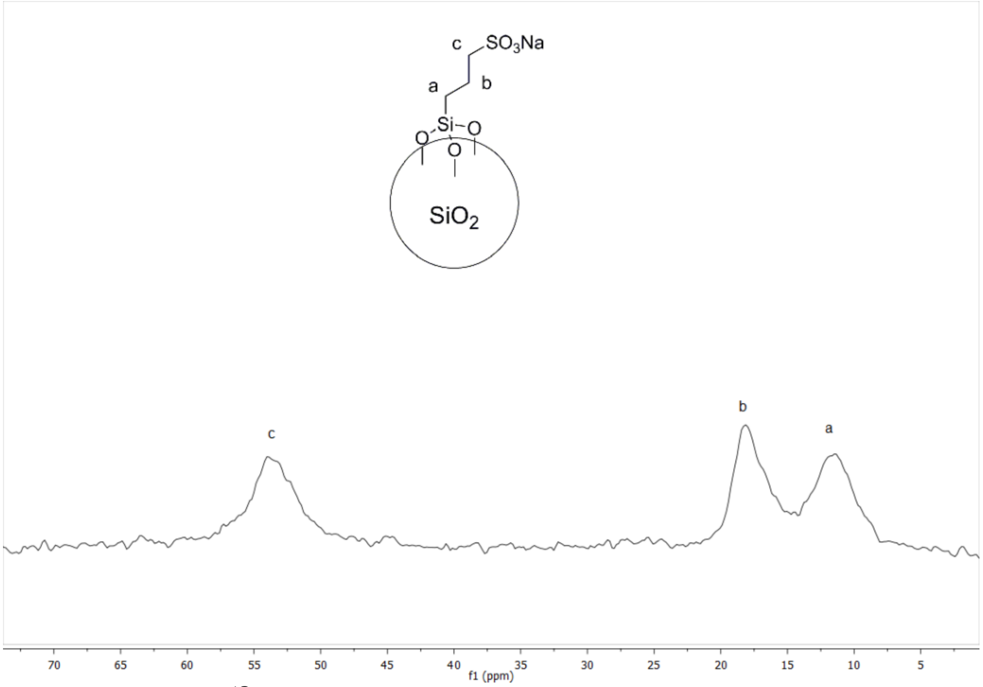

Figure S13. Solid-state ^{13}C NMR spectrum of silica nanoparticles functionalized with sulfonates.

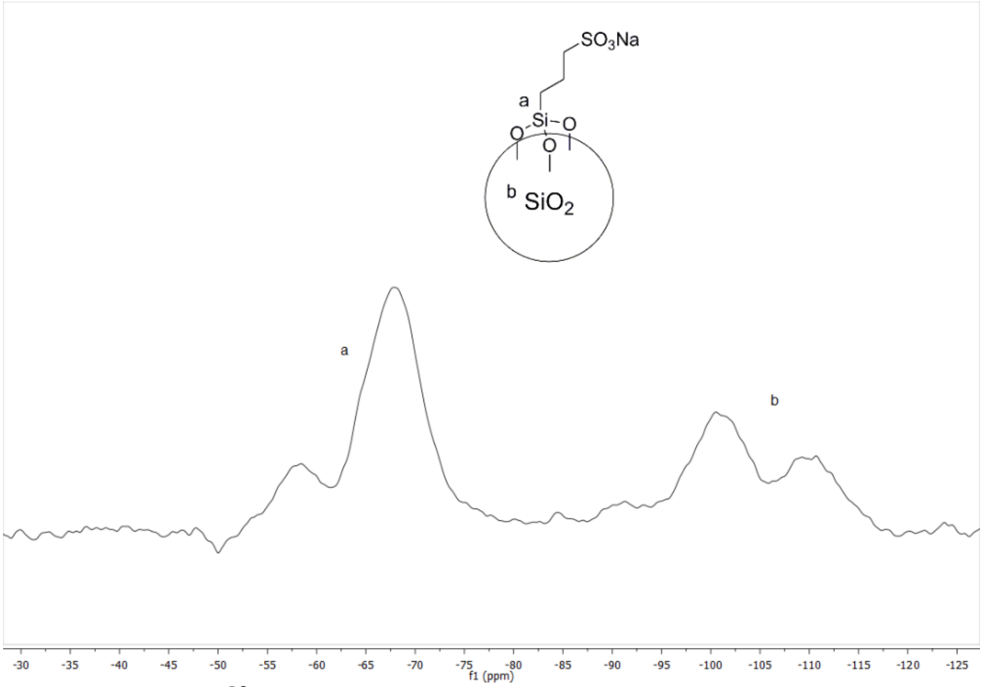

Figure S14. Solid-state ^{29}Si NMR spectrum of silica nanoparticles functionalized with sulfonates.

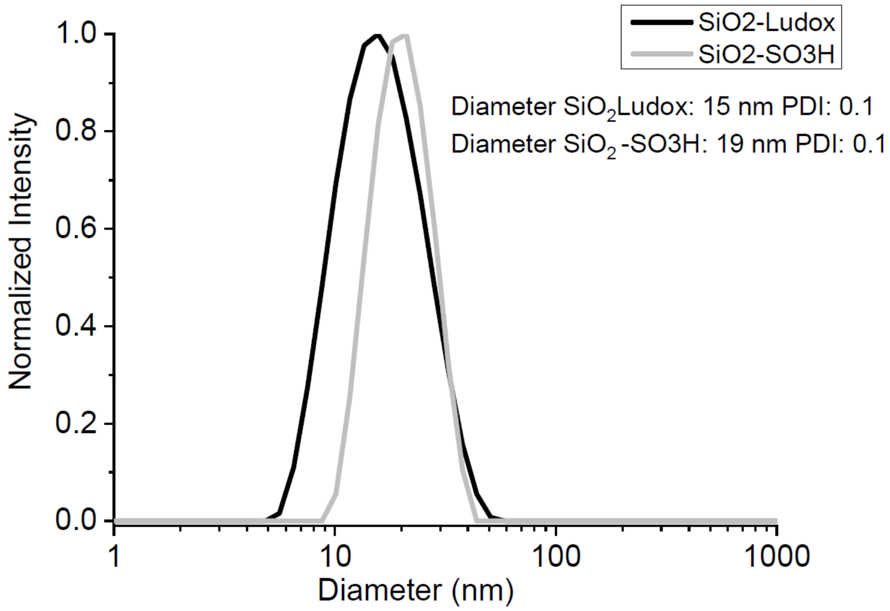

Figure S15. Dynamic light scattering analysis of Ludox HS-30 (hydroxy-functionalized colloidal silica) nanoparticles and the same silica functionalized with sulfonates groups.

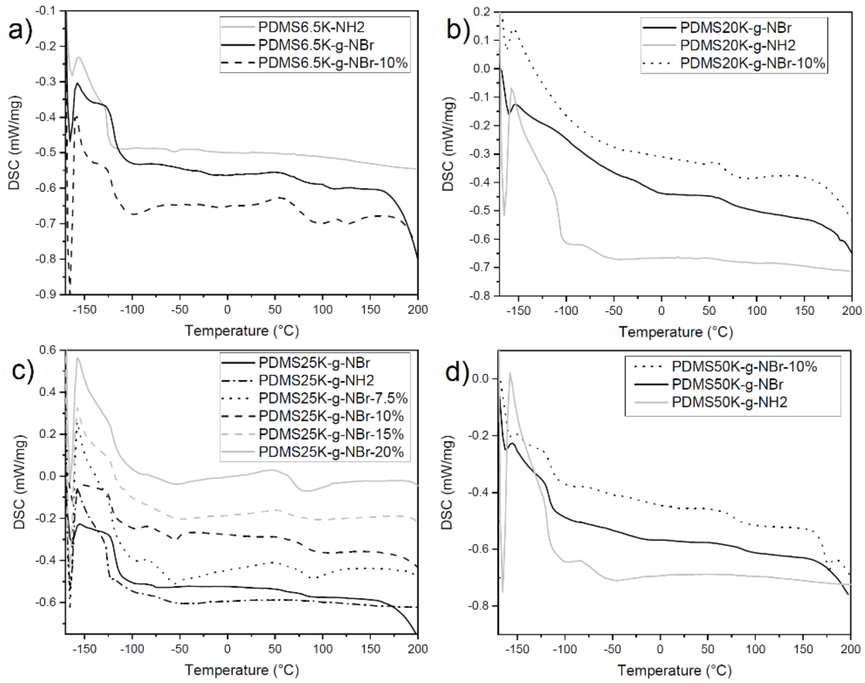

Figure S16. DSC analyses of PDMS-silica nanocomposites of a) PDMS6.5K-g-NBr; b) PDMS20K-g-NBr; c) PDMS25K-g-NBr; and d) PDMS50K-g-NBr.

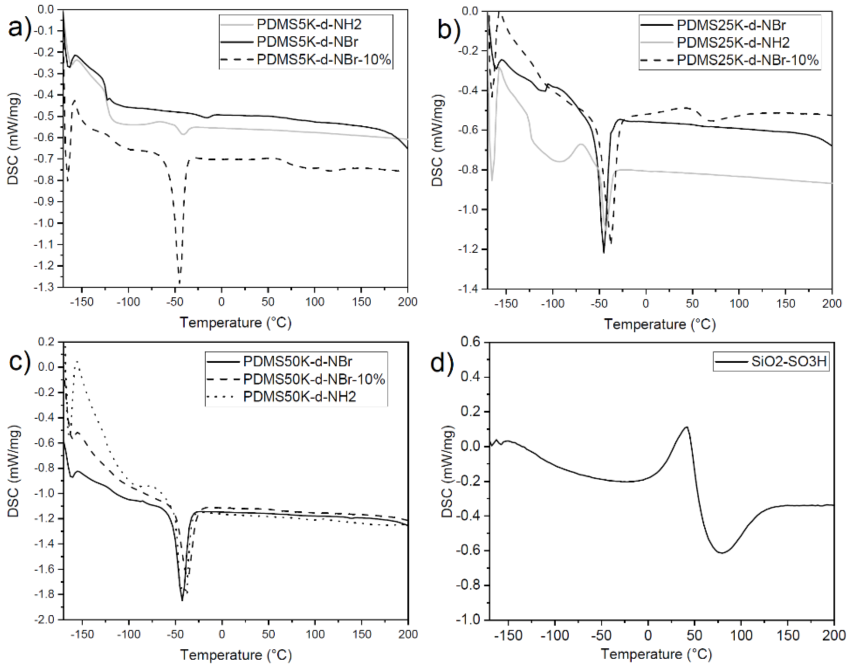

Figure S17. DSC analyses of PDMS-silica nanocomposites of a) PDMS5K-d-NBr; b) PDMS25K-d-NBr; c) PDMS50K-d-NBr; and d) SO_3H-grafted silica.

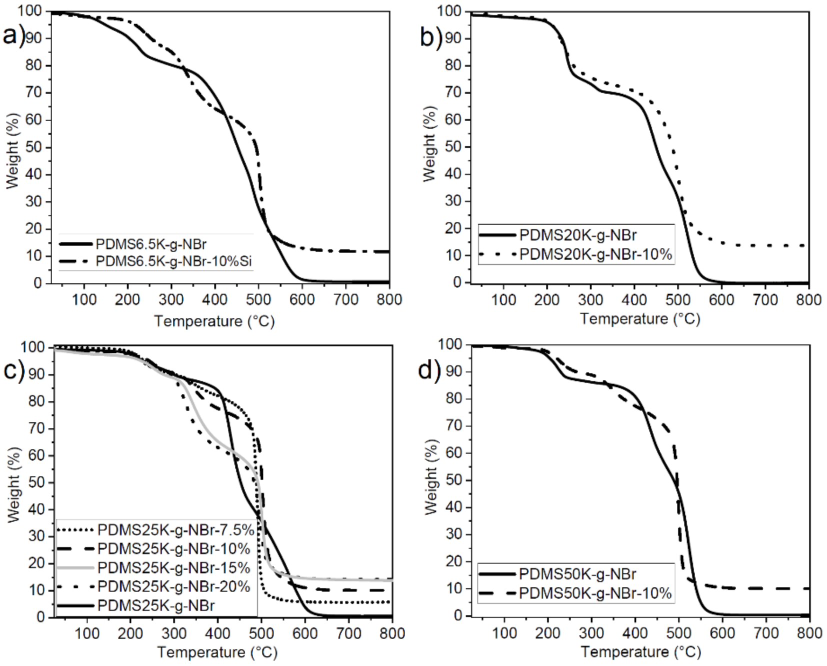

Figure S18. TGA plots of polydimethylsiloxane-silica nanocomposites based on a) PDMS6.5K-g-NBr; b) PDMS20K-g-NBr; c) PDMS25K-g-NBr; and d) PDMS50K-g-NBr.

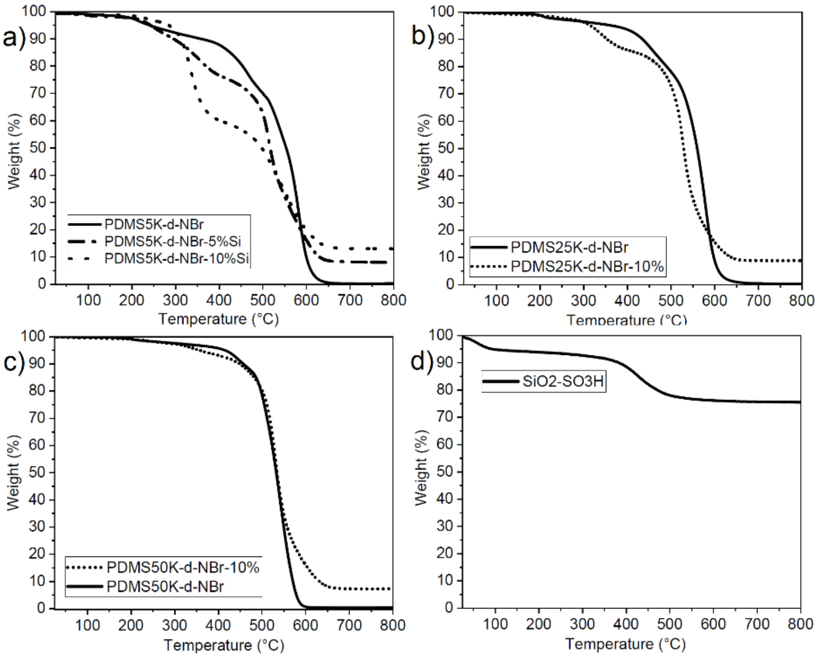

Figure S19. TGA plots of polydimethylsiloxane-silica nanocomposites based on a) PDMS5K-d-NBr; b) PDMS25K-d-NBr; and c) PDMS50K-d-NBr, and TGA plot of d) SO_3H –grafted silica

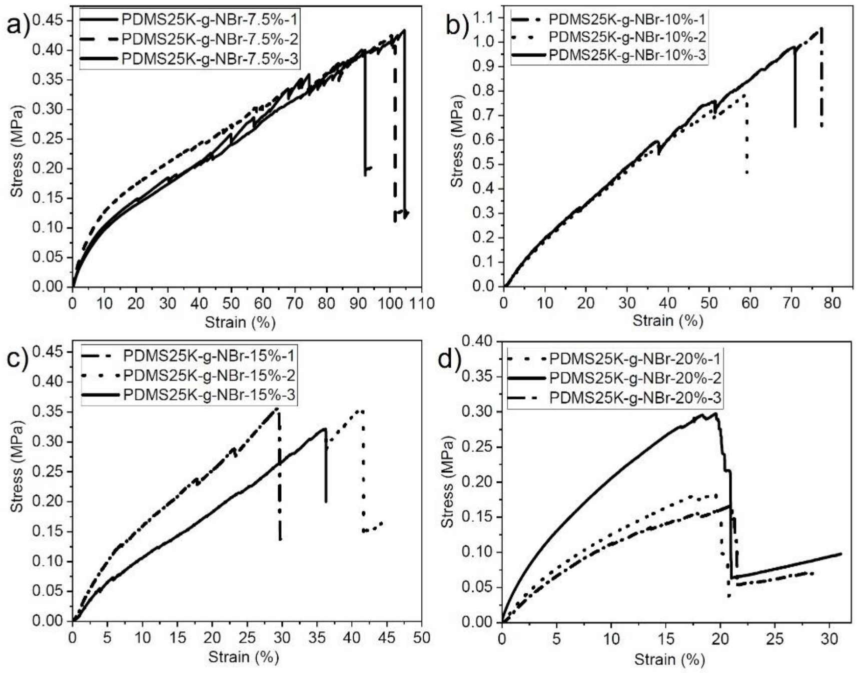

Figure S20. Quasi-static tensile data for the polydimethylsiloxane-silica nanocomposites (10 mm/min) a) PDMS25K-g-NBr-7.5%; b) PDMS25K-g-NBr-10%; c) PDMS25K-NBr-g-15%; and d) PDMS25K-g-NBr-20%.

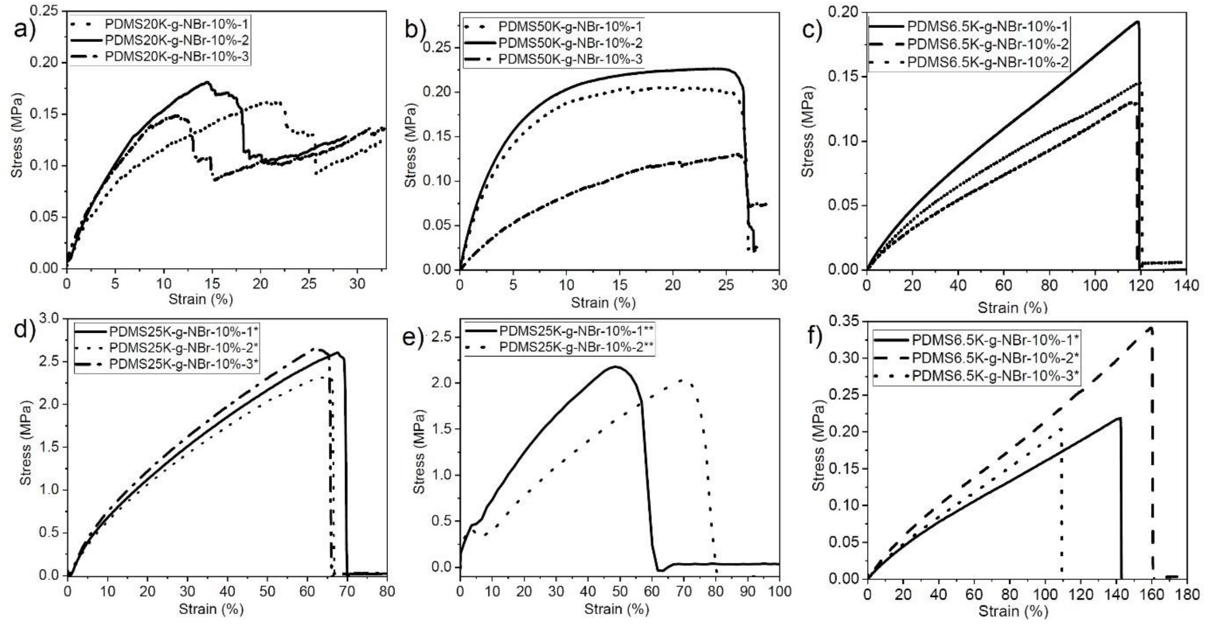

Figure S21. Quasi-static tensile data for the ionic polydimethylsiloxane-silica nanocomposites a) PDMS20K-g-NBr-10% (10 mm/min); b) PDMS50K-g-NBr-10%(10 mm/min); c) PDMS6.5K-g-NBr-10%(10 mm/min); d) PDMS25K-NBr-g-10% (100 mm/min); e) PDMS25K-g-NBr-10% (1000 mm/min); and f) PDMS6.5K-g-NBr-10% (100 mm/min).

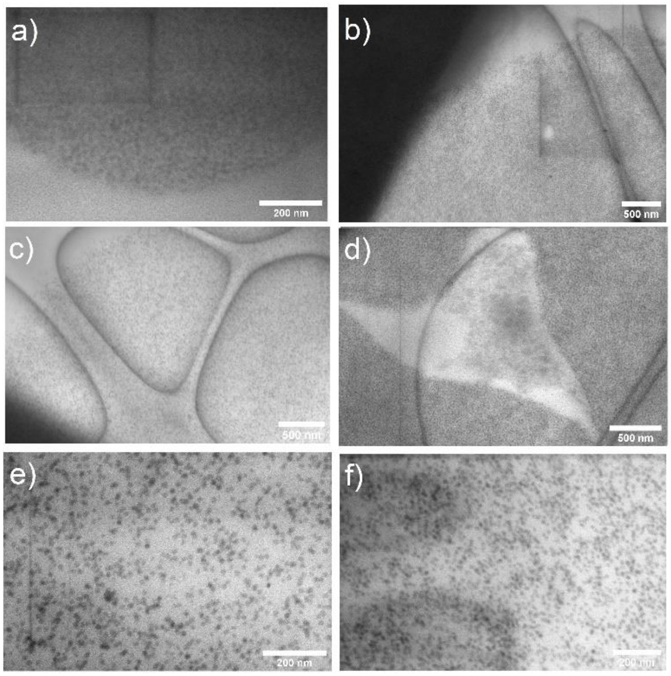

Figure S22. STEM images of a) PDMS20K-g-NBr-5%Si; b) PDMS20K-g-NBr-10%Si; c) PDMS25K-g-NBr-7.5%Si; d) PDMS25K-g-NBr-15%Si; e) PDMS50K-g-NBr-5%Si; and f) PDMS50K-g-NBr-7.5%Si.

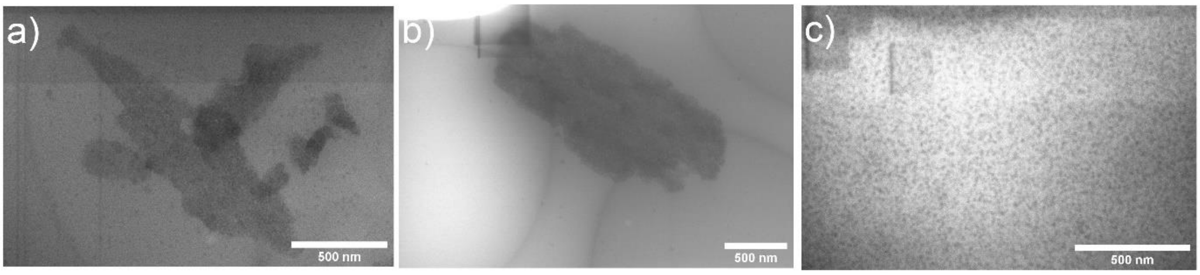

Figure S23. STEM images of a) PDMS25K-d-NBr-5%Si; b) PDMS50K-d-NBr-5%Si; and c) PDMS5K-d-NBr-10%Si.

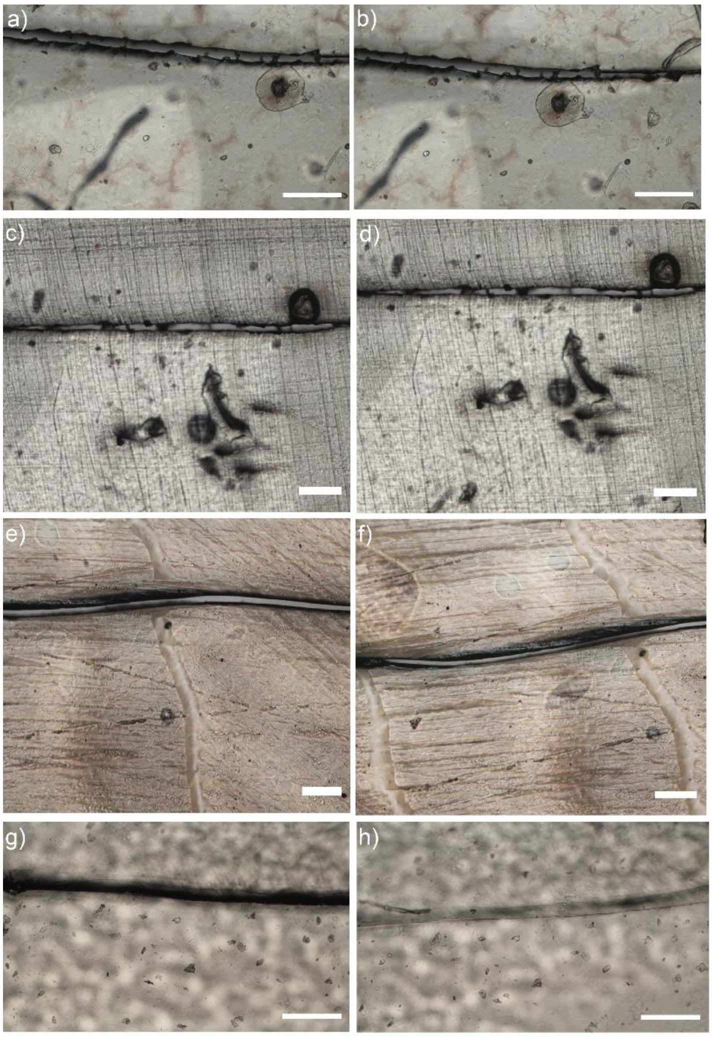

Figure S24. Scratch-healing of the ionic PDMS nanocomposites a) PDMS25K-g-NBr-10wt% and b) PDMS25K-g-NBr-10wt% healed at 80°C for 16 hours; c) PDMS25K-g-NBr-7.5wt% and d) PDMS25K-g-NBr-7.5wt% healed at 80°C for 16 hours; e) PDMS6.5K-g-NBr-10wt% and f) PDMS6.5K-g-NBr-10wt% healed at 80°C for 16 hours; and g) PDMS25K-g-NBr-7.5wt% and h) PDMS25K-g-NBr-7.5wt healed at 80°C in a humid atmosphere for 16 hours. (scale bar 200 nm)

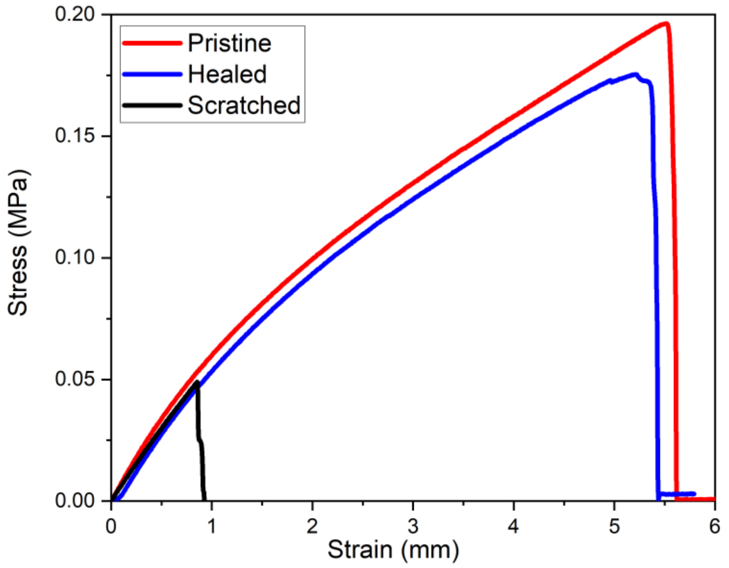

Figure S25. Uniaxial tensile tests of the PDMS6.5K-g-NBr-10% nanocomposites: pristine samples (red), samples after scratch damage (black), and sample after healing of scratches at 80°C under humid atmosphere for an hour.

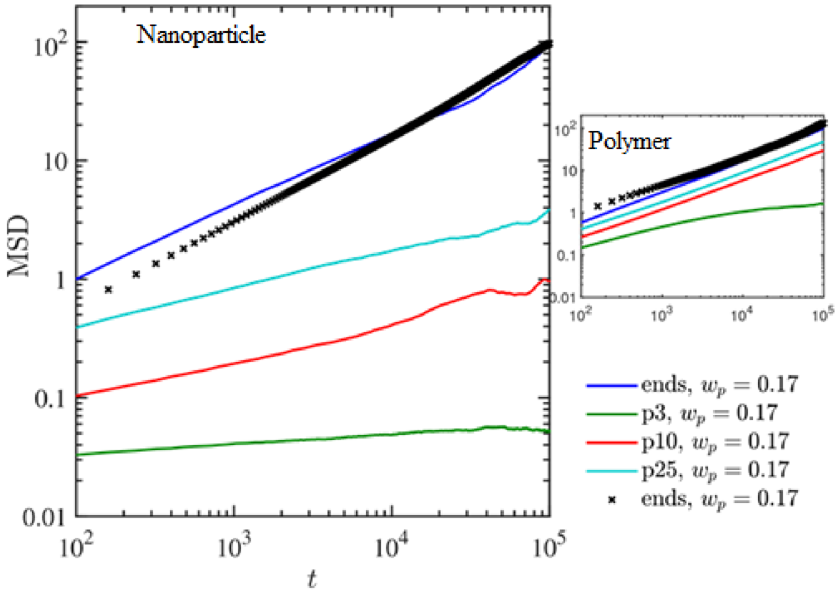

Figure S26. Nanoparticle (main graph) and polymer center of mass dynamics (inset) at w_p=0.17 mass fraction, for different charge densities (either on the backbone or on chain ends). All the solid lines represent nanocomposites where nanoparticles have been dispersed in the matrix (ε_r=24) and black crosses show a nanocomposite with charged ends polymer matrix (ε_r=48), where nanoparticle aggregation was observed.
